# Prognostic impact of *CEBPA* mutational subgroups in adult AML

**DOI:** 10.1038/s41375-024-02140-x

**Published:** 2024-01-16

**Authors:** Julia-Annabell Georgi, Sebastian Stasik, Michael Kramer, Manja Meggendorfer, Christoph Röllig, Torsten Haferlach, Peter Valk, David Linch, Tobias Herold, Nicolas Duployez, Franziska Taube, Jan Moritz Middeke, Uwe Platzbecker, Hubert Serve, Claudia D. Baldus, Carsten Muller-Tidow, Claudia Haferlach, Sarah Koch, Wolfgang E. Berdel, Bernhard J. Woermann, Utz Krug, Jan Braess, Wolfgang Hiddemann, Karsten Spiekermann, Emma L. Boertjes, Robert K. Hills, Alan Burnett, Gerhard Ehninger, Klaus Metzeler, Maja Rothenberg-Thurley, Annika Dufour, Hervé Dombret, Cecile Pautas, Claude Preudhomme, Laurene Fenwarth, Martin Bornhäuser, Rosemary Gale, Christian Thiede

**Affiliations:** 1 Medizinische Klinik und Poliklinik 1, Medizinische Fakultät und Universitätsklinikum Carl Gustav Carus, Technische Universität Dresden, Dresden, Germany; 2 AvenCell Europe GmbH, Dresden, Germany; 3 MLL Münchner Leukämielabor GmbH, Munich, Germany; 4 Erasmus University Medical Center, Rotterdam, Netherlands; 5 Department of Haematology, UCL Cancer Institute, London, UK; 6 Laboratory for Leukemia Diagnostics, Department of Medicine III, University Hospital, LMU Munich, Munich, Germany; 7 Institut de Recherche contre le Cancer de Lille, Centre Hospitalier Universitaire de Lille, Lille, France; 8 Klinik und Poliklinik fur Hämatologie, Zelltherapie und Hämostaseologie, Universitätsklinikum Leipzig, Leipzig, Germany; 9 Medizinische Klinik 2, Universitätsklinikum Frankfurt, Frankfurt am Main, Germany; 10 Klinik für Innere Medizin II, Universitätsklinikum Schleswig-Holstein, Kiel, Germany; 11 Klinik für Hämatologie, Onkologie und Rheumatologie, Universitätsklinikum Heidelberg, Heidelberg, Germany; 12 Department of Medicine A, University Hospital Münster, Münster, Germany; 13 German Society of Hematology and Oncology, Berlin, Germany; 14 Department of Medicine 3, Klinikum Leverkusen, Leverkusen, Germany; 15 Department of Oncology and Hematology, Hospital Barmherzige Brüder, Regensburg, Germany; 16 Department of Medicine III, University Hospital LMU Munich, Munich, Germany; 17 Nuffield Department of Population Health, Oxford University, Oxford, UK; 18 Department of Haematology, Cardiff University, University Hospital of Wales, Cardiff, UK; 19 Hôpital Saint-Louis (AP-HP), EA 3518, Université de Paris, Paris, France; 20 Service d’Hématologie et de thérapie cellulaire, Hôpital Henri Mondor, Créteil, France; 21 Nationales Zentrum für Tumorerkrankungen (NCT), Dresden, Germany; 22 AgenDix GmbH, Dresden, Germany

**Keywords:** Acute myeloid leukaemia, Cancer genetics

## Abstract

Despite recent refinements in the diagnostic and prognostic assessment of *CEBPA* mutations in AML, several questions remain open, i.e. implications of different types of basic region leucin zipper (bZIP) mutations, the role of co-mutations and the allelic state. Using pooled primary data analysis on 1010 *CEBPA*-mutant adult AML patients, a comparison was performed taking into account the type of mutation (bZIP: either typical in-frame insertion/deletion (InDel) mutations (bZIP^InDel^), frameshift InDel or nonsense mutations inducing translational stop (bZIP^STOP^) or single base-pair missense alterations (bZIP^ms^), and transcription activation domain (TAD) mutations) and the allelic state (single (sm*CEBPA*) vs. double mutant (dm*CEBPA*)). Only bZIP^InDel^ patients had significantly higher rates of complete remission and longer relapse free and overall survival (OS) compared with all other *CEBPA-*mutant subgroups. Moreover, co-mutations in bZIP^InDel^ patients (e.g. *GATA2*, *FLT3*, *WT1* as well as ELN2022 adverse risk aberrations) had no independent impact on OS, whereas in non-bZIP^InDel^ patients, grouping according to ELN2022 recommendations added significant prognostic information. In conclusion, these results demonstrate bZIP^InDel^ mutations to be the major independent determinant of outcome in *CEBPA*-mutant AML, thereby refining current classifications according to WHO (including all dm*CEBPA* and sm*CEBPA* bZIP) as well as ELN2022 and ICC recommendations (including *CEBPA* bZIP^ms^).

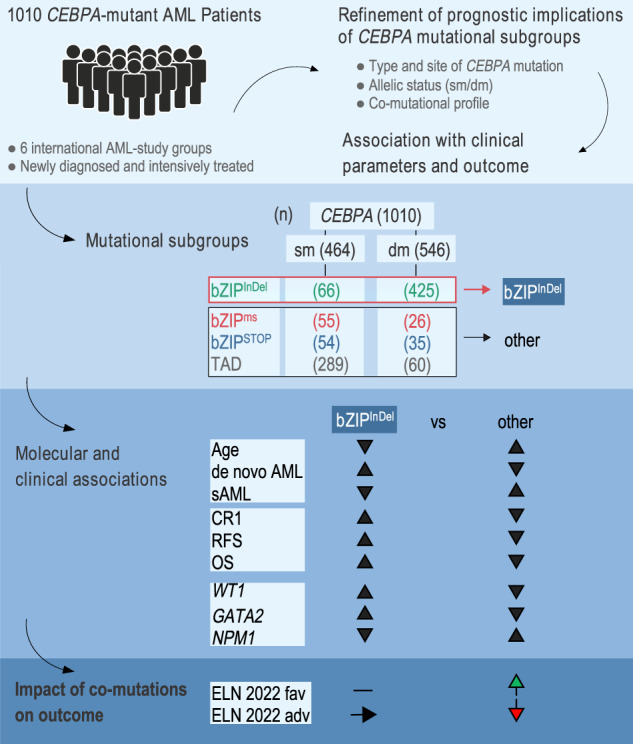

## Introduction

Mutations of the gene encoding the CCAAT-enhancer binding protein alpha (CEBPA) transcription factor are common genetic alterations in acute myeloid leukemia (AML). Since their first description [1], several groups have investigated *CEBPA* mutations in AML (reviewed in [2]). After initial studies suggested that all patients with *CEBPA* mutations carry a more favorable outcome [3–7], subsequent analyses have consistently demonstrated that this improved prognosis is confined to biallelic or double mutations (dm*CEBPA*). Dm*CEBPA* was shown to be associated with a distinct biology and to confer a more favorable clinical outcome, including higher rates of complete remission (CR), reduced relapse risk, and increased overall survival (OS), whereas single allele mutations were considered prognostically irrelevant [8–14]. However, several recent reports looking in more detail for the impact of individual mutations in *CEBPA* suggested that the specific clinical and molecular characteristics as well as the favorable prognosis were restricted to mutations within the basic leucine zipper region (bZIP) region of *CEBPA*, irrespective of their occurrence as double or single mutation [15–17]. Gene expression analysis further supports a unique biology of *CEBPA* bZIP mutations in AML [15, 16]. However, there is evidence that even within this mutational subgroup, biological differences may exist depending on the particular type of *CEBPA* bZIP mutation. Our group observed significant differences in outcome and molecular profiles when comparing patients with in-frame *CEBPA* bZIP mutations (either in-frame insertions/deletions or single base pair missense mutations) and patients with frameshift or nonsense mutations [16].

These findings have provided the basis for a refined biological and clinical classification of *CEBPA* mutations. *CEBPA* bZIP in-frame mutations are now being classified as favorable risk entity in the 2022 update of the European LeukemiaNet (ELN) recommendations on genetic risk classification [18] and in the International Consensus Classification [19]. However, the 2022 WHO classification still defines the *CEBPA* mutational class via the presence of a double mutant allelic status or a single mutant bZIP mutation regardless of the type of mutation(s)/exact DNA alteration [20]. This persistent ambiguity might be due to the fact that few reports have investigated the impact of different *CEBPA* mutational constellations in more detail, so the evidence supporting either of these modifications is still limited.

To gain further insights into the impact of different mutational subtypes, in particular the spectrum of *CEBPA* bZIP mutations, a pooled primary data analysis was performed involving detailed sequencing data as well as clinical variables and treatment outcome from more than 1000 *CEBPA* mutant AML patients. The main objective of this study was to investigate potential differences between different types of bZIP mutations and to examine the relevance of the allelic status (double vs. single mutant).

Our results, generated in the largest cohorts of patients with *CEBPA* mutant AML reported so far, strongly support the notion that the *CEBPA* bZIP^InDel^ genotype introduced in this work (bZIP in-frame insertions/deletions, double and single mutant) shows a specific biology and favorable prognostic implications, whereas the other *CEBPA* mutational subgroups, i.e. TAD mutations as well as bZIP missense and frameshift/nonsense mutations, differ substantially with respect to most clinical as well as molecular factors studied.

This analysis establishes the basis for a more accurate refinement of current classifications and highlights the need for additional research efforts to elucidate the specific biological effects of *CEBPA* bZIP^InDel^ mutations and their role in leukemogenesis.

## Materials/subjects and methods

Anonymized individual patient data from 1010 adult patients aged 16–85 years (median 52 years) were collected from six European AML study groups and registries: *n* = 98 ALFA, *n* = 104 AMLCG, *n* = 191 HOVON, *n* = 200 Munich Leukemia Lab, *n* = 240 SAL, *n* = 177 MRC/NCRI. Patients included were treated between 1989 and 2019, with the majority of individuals treated between 1996 and 2016 (89.4%; Table S1). Patients were treated in prospective trials (details on study protocols are given in the supplement) involving risk stratified post induction therapy according to cytogenetic risk groups, including the option for an allogeneic hematopoietic cell transplantation (alloHCT) in CR1, or recruited to AML registries and biorepositories. Only 40 patients (3.9%) were treated after the release of the ELN 2016 guidelines, which proposed stratification by allelic status (dm*CEBPA* vs. sm*CEBPA*) within the group of *CEBPA*-mutant patients. Otherwise, *CEBPA* mutations were not used for risk stratification in any of the trials. For each individual patient, a predefined minimal data set was collected, including clinical variables, i.e. age, sex, date of AML diagnosis, type of AML (de novo or secondary/therapy-related), bone marrow (BM) blast count, white blood cell (WBC) count, type of and response to induction chemotherapy, date of alloHCT in CR1, date of alloHCT beyond CR1 and events (i.e. induction failure, relapse, death) as well as genetic variables (karyotype, mutational status of *NPM1*, *FLT3*, *GATA2, DNMT3A, IDH1, IDH2, WT1* and other genes, if available). Co-mutational data sufficient for genetic risk stratification according to the ELN2022 guidelines were available for 645 patients (63.8%).

The information collected included complete sequencing results (performed either by Sanger sequencing or next generation sequencing; NGS) of the entire *CEBPA* gene (Genbank Accession No. NM_004364.2). All retrieved *CEBPA* sequences were evaluated for the precise localization of the mutation, i.e. bZIP vs. transcription activation domains [17] 1 and 2, allelic status (sm*CEBPA* vs. dm*CEBPA*) as well as the type of mutation, i.e. insertions/deletions either in-frame or frameshift, missense mutations as well as nonsense mutations.

This study was performed in accordance with the Declaration of Helsinki, all clinical studies and data registries were approved by the local institutional review boards, and written informed consent was obtained from all patients through the participating centers.

For statistical analysis, comparisons of categorical variables between groups were done with the Chi-squared test. Continuous variables were compared with the Kruskal-Wallis-Test between groups. OS was calculated from date of study entry until death, or last follow-up visit. Relapse free survival (RFS) was calculated from date of first remission until date of relapse, date of death, or date of last follow-up visit. Survival endpoints were analyzed with the Kaplan-Meier method. Cox regression models were fitted to estimate hazard ratios. AlloHCT as adjusting variable in multiple models was modeled as time-dependent covariate. Univariate and multivariate logistic regression models were used to estimate odds ratios for achievement of CR1. Individual patient data of the different study groups were pooled. All Cox regression models were stratified for study group. In logistic regression models study group was modeled as a factor. To estimate variability between study groups, all analyses were conducted per study group and interaction of *CEBPA* categories with study group was assessed. Additionally, the estimates from the study groups were pooled via inverse variance method and heterogeneity statistics were estimated. AlloHCT was modeled as time-dependent covariate. A landmark of 3 months from study entry was applied to reduce bias due to early deaths disqualifying patients for alloHCT.

## Results

### CEBPA mutational status

**Fig. 1:**
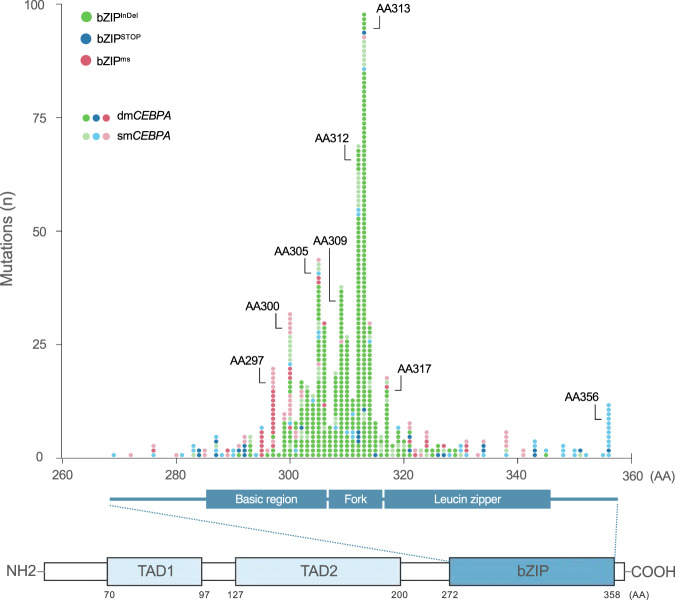
Illustration of type and localization of mutations affecting the bZIP region of the *CEBPA* gene. Mutations in the bZIP region (AA272-358) are typically in-frame insertion or deletion mutations (bZIP^InDel^), whereas frameshift insertions/deletions or nonsense mutations causing a premature translational termination (bZIP^STOP^), and single base-pair missense mutations (bZIP^ms^) are less common.

Of the 1010 patients, 661 patients (65.4%) showed mutations affecting the bZIP-domain of *CEBPA* encompassing amino acids (AA) 272–358 [1]. As illustrated in Fig. 1, mutations in the *CEBPA* bZIP region were typically in-frame insertions or deletions (*n* = 491 pts; 74.3%; referred to as bZIP^InDel^), i.e. (multiples of) 3 bp affecting the DNA-binding-, fork- or bZIP-region. Frameshift insertions/deletions or nonsense mutations in bZIP, causing premature termination of transcription (referred to as bZIP^STOP^), as well as missense mutations (bZIP^ms^), causing single AA changes, were less common and were observed in 81 (12.3%) and 89 (13.5%) of patients, respectively. Interestingly, the different types of mutations showed a non-random distribution, with bZIP^ms^ mainly clustering in certain critical AA positions in the DNA-binding basic region, i.e. AA297 and AA300. In contrast, most bZIP^InDel^ mutations affected the hinge/fork region of the bZIP-domain, i.e., the 14 AAs preceding the first leucine residue of the leucine zipper at position 317 [21], with the most frequent InDel mutations at AA312 and AA313 (Fig. 1). In contrast to the bZIP region, the TAD domains almost uniformly harbored frameshift InDel or nonsense mutations, creating a premature termination codon.

**Fig. 2:**
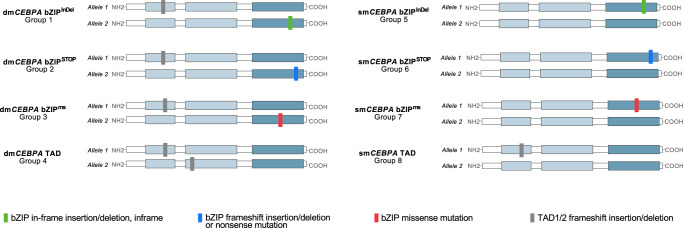
Schematic illustration of the predominant mutational constellations in the eight different *CEBPA*-mutant subgroups. Subgrouping was performed according to different types of mutations, i.e. TAD or bZIP mutations (in-frame insertions/deletions, frameshift insertions/deletions and nonsense mutations, missense mutations) and allelic status (double vs. single mutant).

To assess the impact of different mutational constellations, 8 subgroups were generated, taking into account both type and site of mutation (bZIP^InDel^ vs. bZIP^ms^ vs. bZIP^STOP^ vs. TAD) and allelic status (dm*CEBPA* vs. sm*CEBPA*). The characteristic mutational constellations of the different mutational subgroups (dm*CEBPA* bZIP^InDel^ (Gr1), dm*CEBPA* bZIP^STOP^ (Gr2), dm*CEBPA* bZIP^ms^ (Gr3), dm*CEBPA* TAD (Gr4), sm*CEBPA* bZIP^InDel^ (Gr5), sm*CEBPA* bZIP^STOP^ (Gr6), sm*CEBPA* bZIP^ms^ (Gr7) and sm*CEBPA* TAD (Gr8)) are illustrated in Fig. 2.

In this cohort, patients with dm*CEBPA* predominantly harbored a combination of a bZIP and a TAD mutation (*n* = 475, 87%). In rare cases (*n* = 11), a combination of two bZIP mutations was present, in all of which one of the two mutations was a bZIP^InDel^ mutation. These cases were assigned to Gr1. 60 dm*CEBPA* patients (11%) showed alterations affecting only the TAD regions, most frequently being a combination of TAD1 and TAD2 mutations (Gr4). In 10 patients with dm*CEBPA*, more than two *CEBPA* mutations were detected, including the following combinations: 2 bZIP^InDel^+TAD (assigned to Gr1); bZIP^InDel^+2 TAD (assigned to Gr1, *n* = 2), bZIP^InDel^ + bZIP^ms^+TAD (assigned to Gr1, *n* = 2), bZIP^STOP^+2 TAD (assigned to Gr2); bZIP^ms^+2 TAD (assigned to Gr3), 2 bZIP^ms^+2 TAD (assigned to Gr3), 3 TAD (assigned to Gr4),

### *CEBPA* mutational subgroups and clinical characteristics

**Fig. 3:**
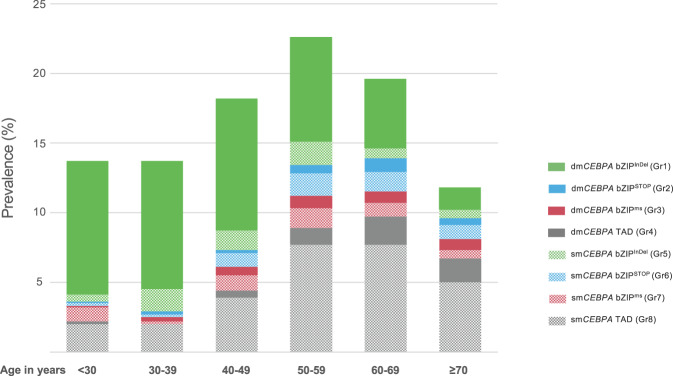
Age distribution of the different *CEBPA*-mutant subgroups. Regarding the distribution of mutational subgroups within age decades, there is a clear decrease in the frequency of *CEBPA* bZIP^InDel^ mutations with increasing age.

As outlined in Table 1, the association of several clinical parameters differed substantially between the defined mutational subgroups. Patients with bZIP^InDel^ mutations, i.e., Gr1 and Gr5, were significantly younger (median age Gr1 42.2 years [IQR 31–54.9]; Gr5 47 years [IQR 39–58]) than those without bZIP^InDel^ mutations (groups 2–4 and groups 6–8) (median age 52–64 years). They also had a higher prevalence of de novo AML (98% and 96%) compared to patients without bZIP^InDel^ mutations (groups 2–4 and groups 6–8), with the latter more frequently evolving as secondary disease after prior MDS or as tAML (rate of de novo AML 81–94%) (Table 1). Categorizing age in 10-year intervals (Fig. 3, Table S2), a continuous decrease in the occurrence of bZIP^InDel^ mutations (especially dm*CEBPA* bZIP^InDel^) was seen with increasing age, whereas bZIP^STOP^ and bZIP^ms^ mutations and alterations affecting only the TAD regions were particularly common in older individuals and less prevalent in patients up to the age of 40 years. Other clinical parameters did not differ significantly between subgroups.

**Table 1:** Clinical and genetic characteristics of the eight different *CEBPA* mutant subgroups.

Parameter	dm*CEBPA* bZIP^InDel^ *n* = 425 Gr1	dm*CEBPA* bZIP^STOP^ *n* = 26 Gr2	dm*CEBPA* bZIP^ms^ *n* = 35 Gr3	dm*CEBPA* TAD *n* = 60 Gr4	sm*CEBPA* bZIP^InDel^ *n* = 66 Gr5	sm*CEBPA* bZIP^STOP^ *n* = 55 Gr6	sm*CEBPA* bZIP^ms^ *n* = 54 Gr7	sm*CEBPA* TAD *n* = 289 Gr8	*p*.value
Age									**<0.001**
median (IQR)	42.4 (31–54.9)	60.5 (54–67.8)	57 (48.5–67.8)	64 (56.08–70)	47 (39–58)	59 (49.85–67)	52 (41–61)	58 (48.6–68)	
Sex									0.507
female, *n*/nval (%)	199/425 (47)	13/26 (50)	17/35 (49)	18/56 (32)	26/65 (40)	24/54 (44)	21/52 (40)	133/283 (47)	
male, *n*/nval (%)	226/425 (53)	13/26 (50)	18/35 (51)	38/56 (68)	39/65 (60)	30/54 (56)	31/52 (60)	150/283 (53)	
AML type									**<0.001**
de novo, *n*/nval (%)	414/423 (98)	21/26 (81)	33/35 (94)	50/57 (88)	61/63 (96)	50/54 (92)	47/52 (90)	253/284 (89)	
sAML, *n*/nval (%)	7/423 (2)	2/26 (8)	1/35 (3)	6/57 (11)	2/63 (4)	4/54 (8)	5/52 (10)	23/284 (8)	
tAML, *n*/nval (%)	2/423 (0)	3/26 (12)	1/35 (3)	1/57 (2)	0/63 (0)	0/54 (0)	0/52 (0)	8/284 (3)	
WBC (Gpt/l)									**<0.001**
median (IQR)	24.1 (9.3–67.5)	6.9 (4.6–16.4)	37.3 (9.1–64.9)	13.2 (4.2–31)	29 (11.1–71.7)	25.5 (2.8–84.3)	17.1 (2.9–60.1)	17.44 (3.9–65.5)	
Bone marrow blasts (%)									**0.010**
median (IQR)	68 (52.5–81.6)	54 (38–73)	64.5 (42–81)	56 (34.5–74.5)	66 (50–80)	67 (48.8–89.3)	71 (50–90)	66.3 (40.3–86)	
Normal karyotype									**0.007**
nval (%)	399 (94)	24 (92)	34 (97)	55 (92)	60 (91)	49 (89)	50 (93)	265 (92)	
nmiss (%)	26 (6)	2 (8)	1 (3)	5 (8)	6 (9)	6 (11)	4 (7)	24 (8)	
No, *n*/nval (%)	92/399 (23)	6/24 (25)	7/34 (21)	20/55 (36)	20/60 (33)	18/49 (37)	23/50 (46)	67/265 (25)	
Yes, *n*/nval (%)	307/399 (77)	18/24 (75)	27/34 (79)	35/55 (64)	40/60 (67)	31/49 (63)	27/50 (54)	198/265 (75)	
ELN 2022^a^									**<0.001**
nval (%)	220 (52)	18 (69)	24 (69)	42 (70)	48 (73)	40 (73)	38 (70)	215 (74)	
nmiss (%)	205 (48)	8 (31)	11 (31)	18 (30)	18 (27)	15 (27)	16 (30)	74 (26)	
favorable, *n*/nval (%)	2/220 (1)	2/18 (11)	2/24 (8)	5/42 (12)	2/48 (4)	5/40 (13)	4/38 (11)	72/215 (33)	
Intermediate, *n*/nval (%)	184/220 (84)	4/18 (22)	14/24 (58)	8/42 (19)	33/48 (69)	10/40 (25)	10/38 (26)	58/215 (27)	
Adverse, *n*/nval (%)	34/220 (15)	12/18 (67)	8/24 (33)	29/42 (69)	13/48 (27)	25/40 (62)	24/38 (63)	85/215 (40)	
CR rate (%)									**<0.001**
median (LCL-UCL)	0.94 (0.92–0.96)	0.74 (0.52–0.9)	0.78 (0.6–0.91)	0.73 (0.59–0.84)	0.92 (0.83–0.97)	0.78 (0.63–0.88)	0.79 (0.66–0.89)	0.74 (0.68–0.79)	
RFS (months)									
median (LCL-UCL)	152 (101.1–NA)	9.4 (5.8–NA)	15 (6.3-NA)	16.4 (9.1–21.5)	63.9 (14.7–NA)	9.2 (7.3–NA)	16.3 (9.6–53.8)	21.8 (16.3–56.3)	**<0.001**
OS (months)									
median (LCL-UCL)	214.9 (148.5–NA)	15.7 (12.4–NA)	39.7 (14.4-NA)	22.3 (16.4–44.1)	126.4 (37.2–NA)	70.9 (12.1–111)	39.7 (14.4–NA)	41.2 (22.4–67.3)	**<0.001**

^a^according to karyotype and comutations, irrespective of *CEBPA* mutational status.

*Gr* group, *IQR* interquartile range, *nval* total number of data points available for evaluation, *nmiss* number of missing data points, *AML* acute myeloid leukemia, *sAML* secondary AML, *tAML* therapy-associated AML, *WBC* white blood cell count, *ELN* European LeukemiaNet, *CR* complete remission, *RFS* relapse-free survival, *OS* overall survival, *LCL* lower confidence level, *ULC* upper confidence level.

### Association of *CEBPA* mutations with other molecular and cytogenetic abnormalities

**Fig. 4:**
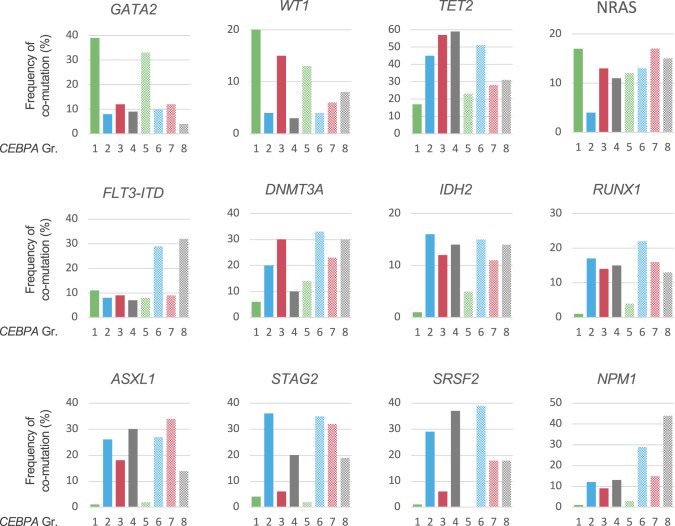
Frequency distribution of additional gene mutations identified in the different *CEBPA*-mutant subgroups. Frequencies are shown for genes with a prevalence of at least 10% in the total *CEBPA-*mutant cohort.

Additional mutations were identified in 861/1010 *CEBPA*-mutant patients (85.2%). Figure 4 illustrates the distribution of co-mutations in the different *CEBPA* mutational subgroups. Significant differences were observed for several genes, the most striking being *GATA2*, which was found mutated in 39 and 33% of patients in Gr1 and Gr5, but only 4–12% in patients carrying non-bZIP^InDel^ mutations (groups 2–4 and 6–8), and *NPM1*, with only 1 and 3% of patients affected in Gr1 and Gr5, but 10–44% in groups 2–4 and 6–8 (*p* < 0.001). In general, the spectrum of co-mutations of patients with bZIP^InDel^ mutations differed markedly from the other *CEBPA* subgroups, the latter more frequently carrying mutations in genes associated with AML after prior MDS, such as spliceosome mutations (i.e. *SRSF2*, *SF3B1*, *U2AF1* or *ZRSR2*) as well as alterations associated with DNA-methylation (i.e. *DNMT3A*, *TET2*, *IDH1* and *IDH2*) (Fig. 4, Table S3). Besides *GATA2*, patients with bZIP^InDel^ mutations were more likely to harbor mutations in *WT1* (Gr1 bZIP^InDel^ 20%, Gr5 13%, all other groups 3–15%).

Most *CEBPA*-mutant patients showed a normal karyotype (Table 1). In patients with bZIP^InDel^ mutations, the predominant cytogenetic aberrations were del 9q (Gr1 *n* = 24/92; Gr5 *n* = 5/20) and +21 (Gr1 *n* = 19/92; Gr5 *n* = 4/20). Among the other *CEBPA* mutational subgroups, chromosomal abnormalities were more diverse and included −7, −5, +8 and several others, thereby reflecting the spectrum of changes seen in AML in general.

### *CEBPA* mutational subgroups and response to treatment

Treatment response data were available for 992 patients (98.2%). Outcome analysis showed comparability of CR rates as well as RFS and OS between study groups (Table S1). Regarding initial response to therapy within the eight different subgroups, patients with bZIP^InDel^ mutations achieved the highest rates of CR1, with 94.3% in Gr1 (OR 6.38 [3.83–10.63], *p* < 0.001) and 92.1% in Gr5 (OR 4.51 [1.71–11.86], *p* = 0.002) compared to CR-rates of 73.1–79.6% observed in the other subgroups (Table 1, Table S4).

**Fig. 5:**
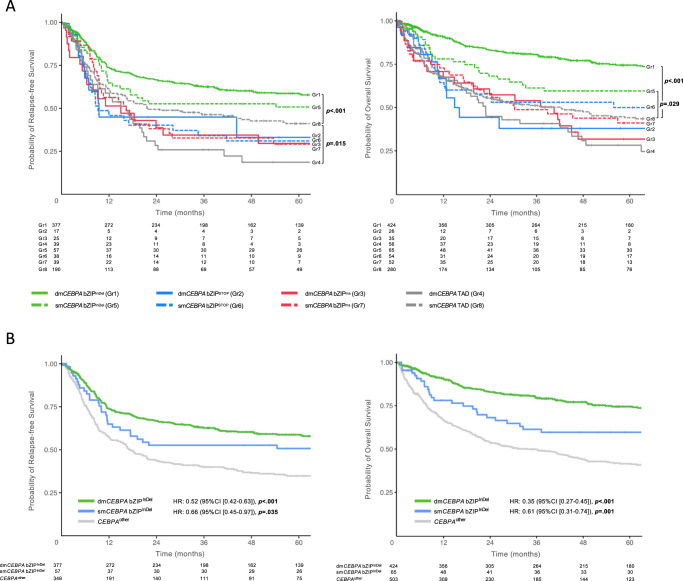
Impact of different *CEBPA*-mutant subgroups on outcome. Kaplan Meyer estimates for RFS and OS in **A** Gr1-8 and **B** Gr1 and Gr5 (dmCEBPA bZIP^InDel^ and smCEBPA bZIP^InDel)^ vs. Gr2-4 and Gr6-8 (CEBPA^other^). HR and *p* values were calculated by Cox regression analysis.

When analyzed according to the eight different mutational subgroups, patients carrying bZIP^InDel^ mutations had a more favorable outcome (RFS and OS) than patients without these mutations (Fig. 5A). In detail, patients in Gr1 showed the longest RFS (median 152 months (HR 0.60 [0.47–0.77]); *p* < 0.001) and OS (median 215 months, HR 0.37 [0.29–0.46], *p* < 0.001). Outcome of Gr5 patients was less favorable than Gr1 (median RFS 64 months (HR 0.77 [0.51–1.15]), *p* = 0.21; median OS 126 months HR = 0.65 [0.44 to 0.96], *p* = 0.029), but still better than for the other subgroups (median RFS ranged between 9.4 and 21.8 months, median OS 15.7 and 70.9 months) (Tables S5 and S6).

In a subsequent analysis combining groups 2–4 and 6–8 as *CEBPA*^other^ (reference group), patients with dm*CEBPA* bZIP^InDel^ as well as sm*CEBPA* bZIP^InDel^ both demonstrated significantly better CR1, RFS and OS compared with *CEBPA*^other^ patients, which was confirmed in multivariate analysis taking into account the individual study groups and the patient age (CR1: dm*CEBPA* bZIP^InDel^ OR 5.82 [3.62–9.36], *p* < 0.001), sm*CEBPA* bZIP^InDel^ OR 4.13 [1.59–10.68], *p* = 0.003; RFS: dm*CEBPA* bZIP^InDel^ HR 0.52 [0.42–0.63], *p* < 0.001, sm*CEBPA* bZIP^InDel^ HR 0.66 [0.45–0.97], *p* = 0.035; OS: dm*CEBPA* bZIP^InDel^ HR 0.35 [0.28–0.43], *p* < 0.001, sm*CEBPA* bZIP^InDel^ HR 0.61 [0.42–0.89], *p* = 0.011 (Fig. 5B, Tables S7–S9).

AlloHCT performed in CR1 showed no benefit in bZIP^InDel^ patients (HR 1.19 [0.81 to 1.75], *p* = 0.178) (Fig. S1, Table S10).

### Impact of co-mutations on outcome in *CEBPA* bZIP^InDel^ mutant patients

Several studies have reported on the effect of co-mutations [22–28] in *CEBPA*-mutant AML. In patients with dm*CEBPA* bZIP^InDel^, a potential concurrent effect on outcome was assessed for the most common alterations previously associated with outcome, i.e. *GATA2*, *TET2*, *WT1*, *CSF3R* and *FLT3-*ITD. The RFS in the few *CSF3R*-mutant patients (*n* = 15) was significantly reduced in univariate analysis (dm*CEBPA* bZIP^InDel^ */CSF3R*^wt^: RFS HR 0.24 [0.14–0.44], *p* < 0.001) (Fig. S2), which remained significant in multivariate analysis (Table S11), however, this effect did not translate into a difference in overall survival (OS HR 1.42 [0.52–3.89]; *p* = 0.491). The presence of a co-mutation in *TET2* was also associated with worse RFS (dm*CEBPA* bZIP^InDel^ */TET2*^wt^: RFS HR 0.61 [0.4–0.95], *p* = 0.028) and OS, (dm*CEBPA* bZIP^InDel^ */TET2*^wt^: OS HR 0.56 [0.34–0.93], *p* = 0.025) (Fig. S3), though this effect lost its significance in multivariate analysis (Table S12). *FLT3*-ITD-positive patients had a shorter RFS in univariate (dm*CEBPA* bZIP^InDel^ without *FLT3-*ITD: RFS HR 0.60 [0.38–0.96], *p* = 0.031) (Fig. S4), but not in multivariate analysis (Table S13). In contrast, the presence of *GATA2* and *WT1* co-mutations did not significantly affect RFS or OS in this group (Figs. S5, S6).

### Impact of ELN2022 adverse molecular and cytogenetic alterations on outcome in *CEBPA* bZIP^InDel^ mutant AML

**Fig. 6:**
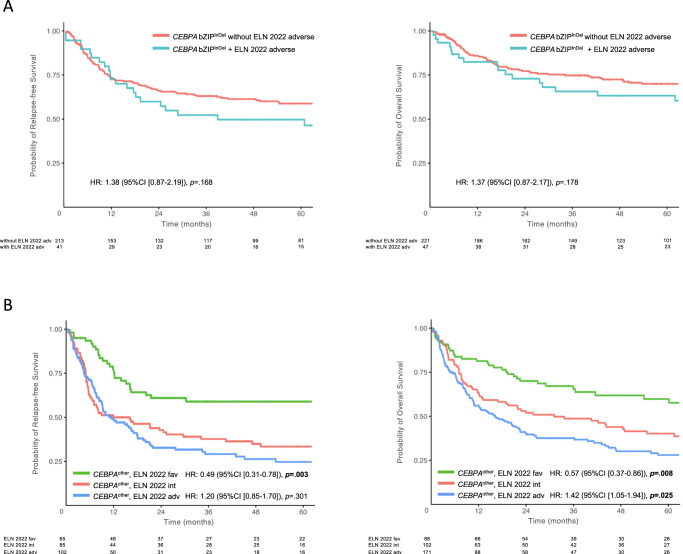
Impact of ELN2022 risk factors in *CEBPA*-mutant subgroups. Kaplan Meyer estimates for RFS and OS of **A** *CEBPA* bZIP^InDel^ patients with vs. without ELN2022 adverse risk factors and **B** different ELN2022 risk groups within *CEBPA*^other^ patients. HR and *p* values were calculated by Cox regression analysis.

To investigate whether cytogenetic and molecular risk factors according to the novel ELN2022 recommendations had an effect on outcome in bZIP^InDel^ patients, further outcome analysis was performed within this subgroup. In total, 48 patients harbored a combination of *CEBPA* bZIP^InDel^ mutations and adverse cytogenetic or molecular abnormalities, including complex karyotypes (*n* = 9), monosomy 5 or 7 (*n* = 6) or mutations in *ASXL1* (*n* = 5), *EZH2* (*n* = 8), *RUNX1* (*n* = 6), *SF3B1* (*n* = 1), *SRSF2* (*n* = 1), *STAG2* (*n* = 8), *TP53* (*n* = 2) and *U2AF1* (*n* = 2). As depicted in Fig. 6A, the presence of ELN2022 adverse genetic factors did not significantly affect the favorable outcome in *CEBPA* bZIP^InDel^-mutant patients (RFS: HR 1.37 [0.87–2.17], *p* = 0.178 and OS: HR 1.46 [0.9–2.37], *p* = 0.129) (Tables S14, S15).

### Impact of ELN2022 mutational subgroups in *CEBPA* mutant patients without bZIP^InDel^ (subgroups 2–4 and 6–8)

Recent analysis suggested that certain co-mutations, in particular mutant *NPM1*, might have an effect in patients with sm*CEBPA* [29]. To gain further insights on the impact of co-mutations in patients without bZIP^InDel^ mutations, i.e. groups 2–4 and 6–8 (*CEBPA*^other^), a combined analysis based on the ELN2022 guidelines was performed for these patients. A total of 345 patients (63.8%) had sufficient cytogenetic and molecular data to allow reclassification according to the ELN2022 risk groups. Most of these patients (*n* = 183/345) (53%) were assigned to the adverse risk group, predominantly due to *ASXL1* (*n* = 85), *RUNX1* (*n* = 63), *SRSF2* (*n* = 56) and *STAG2* (*n* = 55) mutations, while only 27 patients (14.8%) had poor risk cytogenetics.

In the patients re-assigned to the favorable risk group (*n* = 88), all but one patient (showing a t(8;21)) had *NPM1*-mutations. As depicted in Fig. 6B, the outcome of these groups showed statistically significant differences, with the median RFS not reached and a median OS of 154 months in the *CEBPA*^other^/ELN2022 favorable risk group compared to 16 months and 31 months in the *CEBPA*^other^/ELN2022 intermediate risk group and 12 months and 16 months in the *CEBPA*^other^/ELN2022 adverse risk group (*p* < 0.001; for multivariate analysis see Tables S16 and S17).

## Discussion

This study examined the prognostic impact of different *CEBPA* mutational subgroups in detail in a large cohort of patients. The prevalence of mutations in *CEBPA* ranges between 5 and 10 [8], therefore the 1010 *CEBPA*-mutant patients investigated in this study correspond to a total of 10.000–20.000 adult patients with AML. Based on these large numbers, this analysis for the first time allowed us to address several questions which had remained unclear or controversial in previous investigations, namely the impact of missense mutations in the bZIP region, the impact of co-mutations and cytogenetics in patients with bZIP^InDel^ mutations as well as the prognosis of *CEBPA* mutant patients without CEBPA bZIP^InDel^ mutations.

An important conclusion from our study is that it clearly supports the previous findings on the unique behavior of bZIP mutations compared to other types of mutations in *CEBPA*. The results confirm that irrespective of the allelic state, *CEBPA* bZIP^InDel^ mutations define a distinct subgroup characterized by younger age and a specific co-mutational profile, including a high rate of *GATA2* and *WT1* mutations and mutual exclusiveness of other subtype-defining lesions like mutations in *NPM1*. In addition, *CEBPA* bZIP^InDel^ mutations were associated with a very favorable outcome. Patients with bZIP^InDel^ mutations, especially those with double mutant *CEBPA*, demonstrated a 5-year overall survival rate above 75%, indicating that these patients should be considered as one of the AML subgroups with the best response to conventional treatment. In line with this, alloHCT performed in CR1 did not improve outcome in this group. However, relapsed bZIP^InDel^ patients appear to benefit from alloHCT as part of salvage treatment which is most strikingly demonstrated in the small subgroup of patients with *CEBPA* bZIP^InDel^ mutations and *CSF3R* co-mutations, which showed a highly significantly decreased RFS, but no difference in OS due to successful salvage treatment. These results confirm similar observations in pediatric patients [15]. Co-mutations in several other genes have been associated with prognosis in *CEBPA*-mutant AML. In particular, *GATA2* co-mutations were reported to confer a better prognosis in dm*CEBPA* patients by some groups [23, 30], although this was not confirmed by others [22, 24]. The data presented here provide a possible explanation for these discrepant reports. *GATA2* mutations were predominantly found in patients with bZIP^InDel^ mutations, with a significantly lower prevalence in patients with other dm*CEBPA* mutations (Gr.2–4). Given that *GATA2* mutations had no impact on outcome when analysis was restricted to bZIP^InDel^ patients (Fig. S5), this suggests that *GATA2*-mutation status might be a surrogate parameter for bZIP^InDel^-mutations, and therefore associated with better outcome in some studies. The same might be true for several other mutations, i.e., *FLT3* and *TET2*, which have a significantly higher prevalence in non-bZIP^InDel^ patients and had previously been shown to be associated with inferior outcome in *CEBPA*-mutant patients in some studies [23, 28, 31–33]. Very recently *Tet2*-mutations have been demonstrated to enhance aggressiveness of *Cebpa*-mutant disease in animal models [34]. Although significant differences were observed for mutant *TET2* and *FLT3* in univariate analysis, multivariable analysis did not confirm an independent effect of these alterations in bZIP^InDel^ patients. In addition, a combined analysis of adverse molecular and cytogenetic prognostic factors according to ELN 2022 recommendations failed to indicate a significant prognostic impact in the bZIP^InDel^ group. Taken together, these data suggest that *CEBPA* bZIP^InDel^-mutant patients represent a unique subgroup of patients with AML.

In contrast, patients with bZIP^STOP^, bZIP^ms^ or TAD mutations, irrespective of allelic status, showed a different biology and worse outcome. In our previous analysis, bZIP^ms^ mutations were grouped and analyzed together with bZIP^InDel^ as “bZIP in-frame”, which corresponds to the definitions of the current ELN and ICC guidelines [18, 19]. However, in the current analysis of a larger cohort of patients, bZIP^ms^ mutations were clearly associated with an inferior outcome when evaluated separately, and were clinically and molecularly distinct from bZIP^InDel^ mutations, while sharing more commonality with the other *CEBPA* subgroups.

**Fig. 7:**
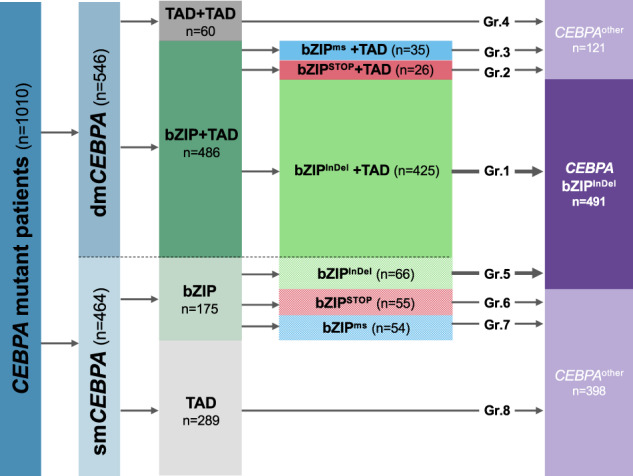
Re-grouping of *CEBPA*-mutant patients according to localization and type of mutation(s). Illustration of the frequency of different *CEBPA* mutational subtypes and their allocation to the proposed subgroups *CEBPA* bZIP^InDel^ and *CEBPA*^other^.

WHO continues to include biallelic *CEBPA* mutations as a defined subgroup [20]. However, our results indicate that patients with dm*CEBPA* without bZIP^InDel^ mutations, i.e. patients showing either two TAD mutations, TAD and bZIP^STOP^ or TAD and bZIP^ms^, differ substantially in biology and outcome, suggesting that only bZIP^InDel^ mutations and not bZIP^ms^ mutations or any other dm*CEBPA* mutations should be included in this specific AML subgroup (Fig. 7). This extends previously published data by El-Sharkawi et al. which already provided evidence for a differential effect of different double mutant constellations [35].

Interestingly, our data indicate that the different *CEBPA* bZIP mutational subtypes, i.e. in-frame InDel mutations, InDel mutations inducing frameshift and missense mutations are distributed in a non-random way in the bZIP region, raising the possibility that mutation location impacts on the functional consequences. As illustrated in Fig. 1, *CEBPA*^InDel^ mutations significantly clustered in the first part of the leucine zipper (between AA310-317) as well as the fork region (AA303-309), whereas missense mutations were significantly more common in the basic region (especially in several highly conserved amino acids, e.g. R300 and R297), which are directly involved in DNA-binding (reviewed in [36]). In contrast, the fork or hinge-region of bZIP-proteins is considered to be especially important for the spacing of the two alpha-helices of the leucine zipper, which in turn could influence either binding specificity and/or affinity of the DNA-binding [21] as well as the interaction with other proteins. *CEBP* proteins bind DNA as homo and heterodimers, and the *CEBPA*-interactome appears to be complex and still not completely understood [37].

Even though the presence of adverse risk aberrations according to the ELN 2022 recommendations was rare in *CEBPA* bZIP^InDel^ mutant patients, knowledge of their prognostic implications is crucial for the choice of post-remission treatment, as it might abrogate the presumed prognostic advantage and low risk of relapse in these AML patients. In this analysis, patients with *CEBPA* bZIP^InDel^ demonstrated superior survival irrespective of concurrent high-risk features. However, it is important to note that the impact of adverse genetic factors according to ELN 2022 in *CEBPA* mutant AML may vary depending on the specific chromosomal or molecular abnormalities.

Aside from *CEBPA* bZIP^InDel^, the other mutational subgroups do not appear to have an independent prognostic value. Analysis based on concomitant cytogenetic and molecular alterations according to current ELN 2022 recommendations within the CEBPA^other^ patient group showed that they conformed to the expected risk stratification group, with no evidence that the *CEBPA* mutation had substantially changed the outcome. For example, a more favorable outcome in these groups was usually attributable to a concomitant *NPM1* mutation.

Although our study represents the largest cohort of *CEBPA*-mutant AML, the analysis also has some limitations, in particular the retrospective nature of the analysis covering a period of almost three decades in which patients were treated. Consequently, none of the patients included were treated with novel targeted agents, e.g. tyrosine kinase inhibitors or Venetoclax/HMA-based therapies, which might affect outcome, at least in subgroups, and the impact of such agents on the different subgroups will be an important issue for future analyses.

In conclusion*, CEBPA* bZIP^InDel^-mutant AML represent a subset of AML with profoundly distinct disease biology and clinical outcomes. Further research efforts aimed at elucidating the underlying molecular mechanisms and identifying additional genetic and epigenetic alterations that interact with *CEBPA* mutations are necessary to harness the full potential of *CEBPA* bZIP^InDel^ mutations in improving the management and prognosis of these AML patients.

### Supplementary information

Supplement

## Data Availability

The datasets generated during and/or analyzed during the current study are available from the corresponding author on reasonable request.
