# Supplementary material for: Prognostic impact of CEBPA mutational subgroups in adult AML
Source: Leukemia. 2024 Jan 16;38(2):281–90. doi: 10.1038/s41375-024-02140-x (PMC10844079; doi:10.1038/s41375-024-02140-x)
Supplement: Supplementary file 1 — Supplement [file 41375_2024_2140_MOESM1_ESM.docx]

Supplement

## Suppl. Table 1 Patient characteristics of the six different study groups

| **Parameter** | **ALFA** n=98 | **AMLCG** n=104 | **HOVON** n=191 | **MLL** n=200 | **MRC** n=177 | **SAL** n=240 |
| --- | --- | --- | --- | --- | --- | --- |
| **Age** |  |  |  |  |  |  |
| median (IQR) | 58 (39-64.8) | 54 (41-66.3) | 53 (39-64) | 58 (42.8-68.3) | 43 (30-53) | 52 (40-63) |
| min - max | 18 - 81 | 18 - 83 | 16 - 77 | 17.8 - 83.2 | 16 - 67 | 17 - 85 |
| **Sex** |  |  |  |  |  |  |
| female, n/nval (%) | 35/98 (36) | 49/104 (47) | 75/177 (42) | 95/200 (48) | 79/177 (45) | 118/240 (49) |
| male, n/nval (%) | 63/98 (64) | 55/104 (53) | 102/177 (58) | 105/200 (52) | 98/177 (55) | 122/240 (51) |
| **AML type** |  |  |  |  |  |  |
| de novo, n/nval (%) | 96/98 (98) | 94/104 (90) | 153/178 (86) | 197/200 (98) | 170/175 (97) | 218/239 (91) |
| sAML, n/nval (%) | 2/98 (2) | 9/104 (9) | 23/178 (13) | 1/200 (0) | 2/175 (1) | 14/239 (6) |
| tAML, n/nval (%) | 0/98 (0) | 1/104 (1) | 2/178 (1) | 2/200 (1) | 3/175 (2) | 7/239 (3) |
| **WBC (Gpt/l)** |  |  |  |  |  |  |
| median (IQR) | 15.4 (4.9-51.6) | 26.2 (6.7-64.7) | 16.6 (4.6-59.3) | 17.5 (5-65.5) | 27.3 (11.2-70.5) | 22 (6.9-64.2) |
| **Bone marrow blasts (%)** |  |  |  |  |  |  |
| median (IQR) | 68 (50-80) | 75 (52-90) | 57 (42-75) | 65 (42.5-80) | 80 (60-91) | 63 (48-78) |
| **Normal karyotype** |  |  |  |  |  |  |
| nmiss (%) | 3 (3) | 3 (3) | 24 (13) | 0 (0) | 17 (10) | 27 (11) |
| No, n/nval (%) | 30/95 (32) | 16/101 (16) | 48/167 (29) | 62/200 (31) | 42/160 (26) | 55/213 (26) |
| Yes, n/nval (%) | 65/95 (68) | 85/101 (84) | 119/167 (71) | 138/200 (69) | 118/160 (74) | 158/213 (74) |
| ***FLT3*-ITD** |  |  |  |  |  |  |
| No, n/nval (%) | 86/98 (88) | 74/100 (74) | 155/189 (82) | 171/200 (86) | 140/177 (79) | 204/240 (85) |
| Yes, n/nval (%) | 12/98 (12) | 26/100 (26) | 34/189 (18) | 29/200 (14) | 37/177 (21) | 36/240 (15) |
| ***NPM1*** |  |  |  |  |  |  |
| No, n/nval (%) | 83/98 (85) | 75/100 (75) | 159/189 (84) | 160/200 (80) | 145/177 (82) | 213/240 (89) |
| Yes, n/nval (%) | 15/98 (15) | 25/100 (25) | 30/189 (16) | 40/200 (20) | 32/177 (18) | 27/240 (11) |
| ***CEBPA*** **subgroup, n/nval (%)** |  |  |  |  |  |  |
| dm*CEBPA* bZIPI^nDel^ | 47/98 (48) | 38/104 (36.5) | 66/191 (34.5) | 80/200 (40) | 83/177 (47) | 111/240 (46.5) |
| dm*CEBPA* bZIP^STOP^ | 4/98 (4)  1/98  7 | 5/104 (5) | 1/191 (0.5) | 6/200 (3) | 2/177 (1) | 8/240 (3.5) |
| dm*CEBPA* bZIP^ms^ | 1/98 (1) | 6/104 (6) | 8/191 (4) | 10/200 (5) | 5/177 (3) | 5/240 (2) |
| dm*CEBPA* TAD | 7/98 (7) | 3/104 (3) | 20/191 (10.5) | 18/200 (9) | 5/177 (3) | 7/240 (3) |
| sm*CEBPA* bZIP^InDel^ | 4/98 (4) | 2/104 (2) | 11/191 (6) | 9/200 (4.5) | 9/177 (5) | 31/240 (13) |
| sm*CEBPA* bZIP^STOP^ | 11/98 (11) | 4/104 (3.5) | 9/191 (5) | 11/200 (5.5) | 10/177 (5.5) | 10/240 (4) |
| sm*CEBPA* bZIP^ms^ | 2/98 (2) | 10/104 (9.5) | 10/191 (5) | 8/200 (4) | 16/177 (9) | 8/240 (3) |
| sm*CEBPA* TAD | 22/98 (23) | 36/104 (34.5) | 66/191 (34.5) | 58/200 (29) | 47/177 (26.5) | 60/240 (25) |
| **CR rate** | 0.816 | 0.808 | 0.915 | 0.767 | 0.864 | 0.846 |
| **5-year RFS** | 0.545 | 0.493 | 0.518 | 0.466 | 0.423 | 0.461 |
| **5-year OS** | 0.546 | 0.559 | 0.579 | 0.601 | 0.640 | 0.502 |
| **Treatment period** |  |  |  |  |  |  |
| until 1990, n/nval (%) | - | - | 4/191(2) | - | 17/177 (9.5) | - |
| 1991-2000, n/nval (%) | - | 13/104 (12.5) | 18/191 (9.5) | - | 75/177(42.5) | 50/240 (21) |
| 2001-2010, n/nval (%) | 34/98 (35) | 62/104 (60) | 48/191 (25) | 101/200 (50.5) | 85/177 (48) | 109/240 (45.5) |
| since 2011, n/nval (%) | 64/98 (75) | 29/104 (27.5) | 121/191(63.5) | 99/200 (49.5) | - | 81/240 (33.5) |

Abbreviations: IQR, interquartile range; nval, total number of data points available for evaluation; nmiss, number of missing data points; AML, acute myeloid leukemia; sAML secondary AML; tAML, therapy-associated AML; WBC, white blood cell count; CR, complete remission; RFS, relapse-free survival; OS, overall surv

## Suppl. Table 2 Distribution of *CEBPA* mutational subgroups in different age decades

|  | **<30** | **30-39** | **40-49** | **50-59** | **60-69** | **≥70** | **p.value** |
| --- | --- | --- | --- | --- | --- | --- | --- |
| **CEBPA subgroups, n/nval (%)** |  |  |  |  |  |  | **<.001** |
| dm*CEBPA* bZIP^InDel^ (Gr1) | 96/137 (70) | 92/137 (67) | 95/182 (52) | 75/226 (33) | 50/196 (26) | 16/118 (14) |  |
| dm*CEBPA* bZIP^STOP^ (Gr2) | 1/137 (1) | 2/137 (1) | 2/182 (1) | 6/226 (3) | 10/196 (5) | 5/118 (4) |  |
| dm*CEBPA* bZIP^ms^ (Gr3) | 1/137 (1) | 3/137 (2) | 6/182 (3) | 9/226 (4) | 8/196 (4) | 8/118 (7) |  |
| dm*CEBPA* TAD (Gr4) | 2/137 (1) | 0/137 (0) | 5/182 (3) | 12/226 (5) | 20/196 (10) | 17/118 (14) |  |
| sm*CEBPA* bZIP^InDel^ (Gr5) | 5/137 (4) | 16/137 (12) | 14/182 (8) | 17/226 (8) | 7/196 (4) | 6/118 (5) |  |
| sm*CEBPA* bZIP^STOP^ (Gr6) | 2/137 (1) | 2/137 (1) | 10/182 (5) | 16/226 (7) | 14/196 (7) | 10/118 (8) |  |
| sm*CEBPA* bZIP^ms^ (Gr7) | 10/137 (7) | 2/137 (1) | 11/182 (6) | 14/226 (6) | 10/196 (5) | 6/118 (5) |  |
| sm*CEBPA* TAD (Gr8) | 20/137 (15) | 20/137 (15) | 39/182 (21) | 77/226 (34) | 77/196 (39) | 50/118 (42) |  |

##

## Suppl. Table 3 Distribution of comutations in eight different *CEBPA* subgroups

| **Parameter** | **dm*CEBPA* bZIP^InDel^** n=425  Gr1 | **dm*CEBPA*bZIP^STOP^** n=26  Gr2 | **dm*CEBPA* bZIP^ms^** n=35  Gr3 | **dm*CEBPA* TAD** n=60  Gr4 | **sm*CEBPA* bZIP^InDel^** n=66  Gr5 | **sm*CEBPA* bZIP^STOP^** n=55  Gr6 | **sm*CEBPA* bZIP^ms^**  n=54  Gr7 | **sm*CEBPA* TAD** n=289  Gr8 | **p.value** |
| --- | --- | --- | --- | --- | --- | --- | --- | --- | --- |
| **GATA2** |  |  |  |  |  |  |  |  | **<.001** |
| nmiss (%) | 24 (6) | 1 (4) | 2 (6) | 2 (3) | 5 (8) | 4 (7) | 2 (4) | 22 (8) |  |
| mut, n/nval (%) | 156/401 (39) | 2/25 (8) | 4/33 (12) | 5/58 (9) | 20/61 (33) | 5/51 (10) | 6/52 (12) | 11/267 (4) |  |
| **ASXL1** |  |  |  |  |  |  |  |  | **<.001** |
| nmiss (%) | 93 (22) | 3 (12) | 7 (20) | 7 (12) | 11 (17) | 10 (18) | 16 (30) | 59 (20) |  |
| mut, n/nval (%) | 4/332 (1) | 6/23 (26) | 5/28 (18) | 16/53 (30) | 1/55 (2) | 12/45 (27) | 13/38 (34) | 33/230 (14) |  |
| **CSF3R** |  |  |  |  |  |  |  |  | .155 |
| nmiss (%) | 36 (8) | 1 (4) | 4 (11) | 3 (5) | 12 (18) | 12 (22) | 17 (31) | 60 (21) |  |
| mut, n/nval (%) | 15/389 (4) | 1/25 (4) | 3/31 (10) | 0/57 (0) | 2/54 (4) | 0/43 (0) | 0/37 (0) | 5/229 (2) |  |
| **CUX1** |  |  |  |  |  |  |  |  | .767 |
| nmiss (%) | 133 (31) | 6 (23) | 7 (20) | 12 (20) | 13 (20) | 21 (38) | 18 (33) | 71 (25) |  |
| mut, n/nval (%) | 8/292 (3) | 0/20 (0) | 1/28 (4) | 2/48 (4) | 0/53 (0) | 0/34 (0) | 1/36 (3) | 4/218 (2) |  |
| **DNMT3A** |  |  |  |  |  |  |  |  | **<.001** |
| nmiss (%) | 17 (4) | 1 (4) | 2 (6) | 2 (3) | 3 (5) | 4 (7) | 1 (2) | 24 (8) |  |
| mut, n/nval (%) | 24/408 (6) | 5/25 (20) | 10/33 (30) | 6/58 (10) | 9/63 (14) | 17/51 (33) | 12/53 (23) | 80/265 (30) |  |
| **EZH2** |  |  |  |  |  |  |  |  | .062 |
| nmiss (%) | 133 (31) | 6 (23) | 7 (20) | 12 (20) | 13 (20) | 21 (38) | 19 (35) | 71 (25) |  |
| mut, n/nval (%) | 7/292 (2) | 3/20 (15) | 0/28 (0) | 3/48 (6) | 2/53 (4) | 0/34 (0) | 2/35 (6) | 7/218 (3) |  |
| **FLT3-ITD** |  |  |  |  |  |  |  |  | **<.001** |
| nmiss (%) | 3 (1) | 0 (0) | 1 (3) | 0 (0) | 0 (0) | 0 (0) | 0 (0) | 2 (1) |  |
| mut, n/nval (%) | 47/422 (11) | 2/26 (8) | 3/34 (9) | 4/60 (7) | 5/66 (8) | 16/55 (29) | 5/54 (9) | 92/287 (32) |  |
| **FLT3-TKD** |  |  |  |  |  |  |  |  | **<.001** |
| nmiss (%) | 4 (1) | 0 (0) | 1 (3) | 0 (0) | 0 (0) | 0 (0) | 0 (0) | 2 (1) |  |
| mut, n/nval (%) | 5/421 (1) | 2/26 (8) | 0/34 (0) | 0/60 (0) | 2/66 (3) | 4/55 (7) | 5/54 (9) | 25/287 (9) |  |
| **IDH1** |  |  |  |  |  |  |  |  | **<.001** |
| nmiss (%) | 30 (7) | 1 (4) | 3 (9) | 2 (3) | 4 (6) | 6 (11) | 5 (9) | 25 (9) |  |
| mut, n/nval (%) | 5/395 (1) | 4/25 (16) | 1/32 (3) | 3/58 (5) | 1/62 (2) | 3/49 (6) | 1/49 (2) | 26/264 (10) |  |
| **IDH2** |  |  |  |  |  |  |  |  | **<.001** |
| nmiss (%) | 12 (3) | 1 (4) | 2 (6) | 2 (3) | 2 (3) | 2 (4) | 0 (0) | 11 (4) |  |
| mut, n/nval (%) | 5/413 (1) | 4/25 (16) | 4/33 (12) | 8/58 (14) | 3/64 (5) | 8/53 (15) | 6/54 (11) | 38/278 (14) |  |
| **KIT** |  |  |  |  |  |  |  |  | .060 |
| nmiss (%) | 132 (31) | 3 (12) | 12 (34) | 10 (17) | 16 (24) | 18 (33) | 20 (37) | 79 (27) |  |
| mut, n/nval (%) | 12/293 (4) | 2/23 (9) | 2/23 (9) | 1/50 (2) | 3/50 (6) | 0/37 (0) | 1/34 (3) | 1/210 (0) |  |
| **KRAS** |  |  |  |  |  |  |  |  | .066 |
| nmiss (%) | 68 (16) | 3 (12) | 5 (14) | 5 (8) | 8 (12) | 9 (16) | 13 (24) | 41 (14) |  |
| mut, n/nval (%) | 11/357 (3) | 0/23 (0) | 1/30 (3) | 1/55 (2) | 0/58 (0) | 2/46 (4) | 5/41 (12) | 9/248 (4) |  |
| **NPM1** |  |  |  |  |  |  |  |  | **<.001** |
| nmiss (%) | 3 (1) | 0 (0) | 1 (3) | 0 (0) | 0 (0) | 0 (0) | 0 (0) | 2 (1) |  |
| mut, n/nval (%) | 3/422 (1) | 3/26 (12) | 3/34 (9) | 8/60 (13) | 2/66 (3) | 16/55 (29) | 8/54 (15) | 126/287 (44) |  |
| **NRAS** |  |  |  |  |  |  |  |  | .703 |
| nmiss (%) | 61 (14) | 2 (8) | 4 (11) | 4 (7) | 9 (14) | 8 (15) | 12 (22) | 39 (13) |  |
| mut, n/nval (%) | 61/364 (17) | 1/24 (4) | 4/31 (13) | 6/56 (11) | 7/57 (12) | 6/47 (13) | 7/42 (17) | 37/250 (15) |  |
| **PDGFRA** |  |  |  |  |  |  |  |  | .230 |
| nmiss (%) | 213 (50) | 12 (46) | 17 (49) | 30 (50) | 22 (33) | 32 (58) | 26 (48) | 129 (45) |  |
| mut, n/nval (%) | 1/212 (0) | 0/14 (0) | 0/18 (0) | 1/30 (3) | 1/44 (2) | 0/23 (0) | 2/28 (7) | 3/160 (2) |  |
| **PHF6** |  |  |  |  |  |  |  |  | .581 |
| nmiss (%) | 213 (50) | 12 (46) | 17 (49) | 30 (50) | 22 (33) | 32 (58) | 26 (48) | 129 (45) |  |
| mut, n/nval (%) | 2/212 (1) | 0/14 (0) | 1/18 (6) | 0/30 (0) | 0/44 (0) | 0/23 (0) | 1/28 (4) | 3/160 (2) |  |
| **PTPN11** |  |  |  |  |  |  |  |  | .114 |
| nmiss (%) | 213 (50) | 12 (46) | 17 (49) | 30 (50) | 22 (33) | 32 (58) | 26 (48) | 129 (45) |  |
| mut, n/nval (%) | 1/212 (0) | 0/14 (0) | 0/18 (0) | 0/30 (0) | 0/44 (0) | 1/23 (4) | 2/28 (7) | 5/160 (3) |  |
| **RAD21** |  |  |  |  |  |  |  |  | .743 |
| nmiss (%) | 213 (50) | 12 (46) | 17 (49) | 30 (50) | 22 (33) | 32 (58) | 26 (48) | 129 (45) |  |
| mut, n/nval (%) | 6/212 (3) | 1/14 (7) | 1/18 (6) | 1/30 (3) | 2/44 (5) | 0/23 (0) | 1/28 (4) | 2/160 (1) |  |
| **RUNX1** |  |  |  |  |  |  |  |  | **<.001** |
| nmiss (%) | 87 (20) | 2 (8) | 7 (20) | 5 (8) | 10 (15) | 10 (18) | 16 (30) | 54 (19) |  |
| mut, n/nval (%) | 5/338 (1) | 4/24 (17) | 4/28 (14) | 8/55 (15) | 2/56 (4) | 10/45 (22) | 6/38 (16) | 31/235 (13) |  |
| **SETBP1** |  |  |  |  |  |  |  |  | **<.001** |
| nmiss (%) | 213 (50) | 12 (46) | 17 (49) | 30 (50) | 22 (33) | 32 (58) | 26 (48) | 129 (45) |  |
| mut, n/nval (%) | 0/212 (0) | 0/14 (0) | 0/18 (0) | 0/30 (0) | 0/44 (0) | 0/23 (0) | 0/28 (0) | 0/160 (0) |  |
| **SF3B1** |  |  |  |  |  |  |  |  | .327 |
| nmiss (%) | 213 (50) | 12 (46) | 17 (49) | 30 (50) | 22 (33) | 32 (58) | 26 (48) | 129 (45) |  |
| mut, n/nval (%) | 0/212 (0) | 0/14 (0) | 0/18 (0) | 0/30 (0) | 1/44 (2) | 0/23 (0) | 1/28 (4) | 4/160 (2) |  |
| **SMC1A** |  |  |  |  |  |  |  |  | **.001** |
| nmiss (%) | 213 (50) | 12 (46) | 17 (49) | 30 (50) | 22 (33) | 32 (58) | 26 (48) | 129 (45) |  |
| mut, n/nval (%) | 0/212 (0) | 2/14 (14) | 1/18 (6) | 1/30 (3) | 0/44 (0) | 0/23 (0) | 0/28 (0) | 4/160 (2) |  |
| **SMC3** |  |  |  |  |  |  |  |  | .051 |
| nmiss (%) | 213 (50) | 12 (46) | 17 (49) | 30 (50) | 22 (33) | 32 (58) | 26 (48) | 129 (45) |  |
| mut, n/nval (%) | 0/212 (0) | 0/14 (0) | 0/18 (0) | 0/30 (0) | 0/44 (0) | 0/23 (0) | 0/28 (0) | 6/160 (4) |  |
| **SRSF2** |  |  |  |  |  |  |  |  | **<.001** |
| nmiss (%) | 213 (50) | 12 (46) | 17 (49) | 30 (50) | 22 (33) | 32 (58) | 26 (48) | 129 (45) |  |
| mut, n/nval (%) | 2/212 (1) | 4/14 (29) | 1/18 (6) | 11/30 (37) | 0/44 (0) | 9/23 (39) | 5/28 (18) | 29/160 (18) |  |
| **STAG2** |  |  |  |  |  |  |  |  | **<.001** |
| nmiss (%) | 213 (50) | 12 (46) | 17 (49) | 30 (50) | 22 (33) | 32 (58) | 26 (48) | 129 (45) |  |
| mut, n/nval (%) | 8/212 (4) | 5/14 (36) | 1/18 (6) | 6/30 (20) | 1/44 (2) | 8/23 (35) | 9/28 (32) | 30/160 (19) |  |
| **TET2** |  |  |  |  |  |  |  |  | **<.001** |
| nmiss (%) | 70 (16) | 4 (15) | 5 (14) | 4 (7) | 6 (9) | 8 (15) | 8 (15) | 41 (14) |  |
| mut, n/nval (%) | 60/355 (17) | 10/22 (45) | 17/30 (57) | 33/56 (59) | 14/60 (23) | 24/47 (51) | 13/46 (28) | 78/248 (31) |  |
| **TP53** |  |  |  |  |  |  |  |  | .058 |
| nmiss (%) | 99 (23) | 3 (12) | 8 (23) | 7 (12) | 12 (18) | 13 (24) | 18 (33) | 70 (24) |  |
| mut, n/nval (%) | 0/326 (0) | 0/23 (0) | 0/27 (0) | 1/53 (2) | 2/54 (4) | 1/42 (2) | 2/36 (6) | 4/219 (2) |  |
| **U2AF1** |  |  |  |  |  |  |  |  | .175 |
| nmiss (%) | 213 (50) | 12 (46) | 17 (49) | 30 (50) | 22 (33) | 32 (58) | 26 (48) | 129 (45) |  |
| mut, n/nval (%) | 1/212 (0) | 0/14 (0) | 0/18 (0) | 0/30 (0) | 1/44 (2) | 0/23 (0) | 2/28 (7) | 3/160 (2) |  |
| **WT1** |  |  |  |  |  |  |  |  | **<.001** |
| nmiss (%) | 22 (5) | 1 (4) | 2 (6) | 1 (2) | 3 (5) | 2 (4) | 1 (2) | 15 (5) |  |
| mut, n/nval (%) | 82/403 (20) | 1/25 (4) | 5/33 (15) | 2/59 (3) | 8/63 (13) | 2/53 (4) | 3/53 (6) | 23/274 (8) |  |
| **ZRSR2** |  |  |  |  |  |  |  |  | .051 |
| nmiss (%) | 213 (50) | 12 (46) | 17 (49) | 30 (50) | 22 (33) | 32 (58) | 26 (48) | 129 (45) |  |
| mut, n/nval (%) | 0/212 (0) | 0/14 (0) | 0/18 (0) | 0/30 (0) | 0/44 (0) | 0/23 (0) | 0/28 (0) | 6/160 (4) |  |

##

## Suppl. Table 4 (A) Univariate and (B) multivariate logistic regression model for CR1 in eight different *CEBPA* subgroups.

**(A)**

| **Parameter** | **OR** | **95%CI** | **p-value** |
| --- | --- | --- | --- |
| dm*CEBPA* bZIP^InDel^ (Gr1) | 6.382 | (3.834 to 10.625) | **<0.001** |
| dm*CEBPA* bZIP^STOP^ (Gr2) | 1.222 | (0.458 to 3.263) | 0.688 |
| dm*CEBPA* bZIP^ms^ (Gr3) | 1.318 | (0.536 to 3.239) | 0.548 |
| dm*CEBPA* TAD (Gr4) | 0.997 | (0.497 to 2) | 0.994 |
| sm*CEBPA* bZIP^InDel^ (Gr5) | 4.509 | (1.714 to 11.862) | **0.002** |
| sm*CEBPA* bZIP^STOP^ (Gr6) | 1.334 | (0.636 to 2.797) | 0.445 |
| sm*CEBPA* bZIP^ms^ (Gr7) | 1.409 | (0.659 to 3.013) | 0.377 |

## (B)

| **Parameter** | **OR** | **95%CI** | **p-value** |
| --- | --- | --- | --- |
| dm*CEBPA* bZIP^InDel^ (Gr1) | 9.345 | (4.898 to 17.827) | **<0.001** |
| dm*CEBPA* bZIP^STOP^ (Gr2) | 2.549 | (0.449 to 14.486) | 0.291 |
| dm*CEBPA* bZIP^ms^ (Gr3) | 1.958 | (0.73 to 5.251) | 0.182 |
| dm*CEBPA* TAD (Gr4) | 1.719 | (0.777 to 3.802) | 0.181 |
| sm*CEBPA* bZIP^InDel^ (Gr5) | 6.211 | (2.198 to 17.555) | **0.001** |
| sm*CEBPA* bZIP^STOP^ (Gr6) | 1.725 | (0.716 to 4.156) | 0.225 |
| sm*CEBPA* bZIP^ms^ (Gr7) | 2.064 | (0.798 to 5.336) | 0.135 |
| AGE | 0.974 | (0.958 to 0.99) | **0.002** |
| AMLSTATsAML | 0.505 | (0.229 to 1.114) | 0.091 |
| AMLSTATtAML | 0.595 | (0.153 to 2.314) | 0.454 |
| I(log(WBC, 2)) | 0.811 | (0.728 to 0.904) | **<0.001** |
| FLT3I | 0.67 | (0.339 to 1.324) | 0.249 |
| NPM1 | 2.803 | (1.35 to 5.82) | **0.006** |

##

## Suppl. Table 5 (A) Cox regression model and (B) multiple cox regression model for RFS in eight different *CEBPA* subgroups.

## (A)

| **Parameter** | **HR** | **95%CI** | **p** |
| --- | --- | --- | --- |
| dm*CEBPA* bZIP^InDel^ (Gr1) | 0.601 | (0.471 to 0.767) | **<0.001** |
| dm*CEBPA* bZIP^STOP^ (Gr2) | 1.264 | (0.641 to 2.496) | 0.498 |
| dm*CEBPA* bZIP^ms^ (Gr3) | 1.456 | (0.884 to 2.397) | 0.139 |
| dm*CEBPA* TAD (Gr4) | 1.633 | (1.101 to 2.422) | **0.015** |
| sm*CEBPA* bZIP^InDel^ (Gr5) | 0.771 | (0.514 to 1.154) | 0.205 |
| sm*CEBPA* bZIP^STOP^ (Gr6) | 1.306 | (0.846 to 2.017) | 0.229 |
| sm*CEBPA* bZIP^ms^ (Gr7) | 1.247 | (0.823 to 1.889) | 0.297 |

## (B)

| **Parameter** | **HR** | **95%CI** | **p** |
| --- | --- | --- | --- |
| dm*CEBPA* bZIP^InDel^ (Gr1) | 0.472 | (0.343 to 0.651) | **<0.001** |
| dm*CEBPA* bZIP^STOP^ (Gr2) | 1.081 | (0.54 to 2.164) | 0.826 |
| dm*CEBPA* bZIP^ms^ (Gr3) | 1.053 | (0.607 to 1.828) | 0.853 |
| dm*CEBPA* TAD (Gr4) | 1.148 | (0.748 to 1.761) | 0.527 |
| sm*CEBPA* bZIP^InDel^ (Gr5) | 0.553 | (0.344 to 0.888) | **0.014** |
| sm*CEBPA* bZIP^STOP^ (Gr6) | 1.128 | (0.691 to 1.84) | 0.631 |
| sm*CEBPA* bZIP^ms^ (Gr7) | 1.072 | (0.658 to 1.747) | 0.782 |
| AGE | 1.021 | (1.013 to 1.029) | **<0.001** |
| AMLSTATsAML | 1.522 | (0.99 to 2.338) | 0.055 |
| AMLSTATtAML | 1.026 | (0.375 to 2.802) | 0.961 |
| I(log(WBC, 2)) | 1.146 | (1.083 to 1.212) | **<0.001** |
| FLT3I | 1.32 | (0.913 to 1.907) | 0.141 |
| NPM1 | 0.389 | (0.248 to 0.611) | **<0.001** |
| alSCTCR1 | 0.798 | (0.604 to 1.054) | 0.111 |

## Suppl. Table 6 (A) Cox regression model and (B) multiple cox regression model for OS in eight different *CEBPA* subgroups.

## (A)

| **Parameter** | **HR** | **95%CI** | **p** |
| --- | --- | --- | --- |
| dm*CEBPA* bZIP^InDel^ (Gr1) | 0.366 | (0.289 to 0.464) | **<0.001** |
| dm*CEBPA* bZIP^STOP^ (Gr2) | 1.213 | (0.688 to 2.137) | 0.504 |
| dm*CEBPA* bZIP^ms^ (Gr3) | 1.186 | (0.759 to 1.852) | 0.451 |
| dm*CEBPA* TAD (Gr4) | 1.41 | (0.999 to 1.988) | 0.051 |
| sm*CEBPA* bZIP^InDel^ (Gr5) | 0.648 | (0.438 to 0.957) | **0.029** |
| sm*CEBPA* bZIP^STOP^ (Gr6) | 0.895 | (0.604 to 1.324) | 0.579 |
| sm*CEBPA* bZIP^ms^ (Gr7) | 1.045 | (0.726 to 1.505) | 0.813 |

## (B)

| **Parameter** | **HR** | **95%CI** | **p** |
| --- | --- | --- | --- |
| dm*CEBPA* bZIP^InDel^ (Gr1) | 0.346 | (0.257 to 0.467) | **<0.001** |
| dm*CEBPA* bZIP^STOP^ (Gr2) | 0.931 | (0.567 to 1.527) | 0.775 |
| dm*CEBPA* bZIP^ms^ (Gr3) | 1.026 | (0.564 to 1.866) | 0.932 |
| dm*CEBPA* TAD (Gr4) | 0.977 | (0.676 to 1.413) | 0.903 |
| sm*CEBPA* bZIP^InDel^ (Gr5) | 0.477 | (0.303 to 0.751) | **0.001** |
| sm*CEBPA* bZIP^STOP^ (Gr6) | 0.916 | (0.596 to 1.409) | 0.689 |
| sm*CEBPA* bZIP^ms^ (Gr7) | 1.103 | (0.717 to 1.698) | 0.656 |
| AGE | 1.03 | (1.022 to 1.038) | **<0.001** |
| AMLSTATsAML | 1.878 | (1.315 to 2.683) | 0.001 |
| AMLSTATtAML | 1.271 | (0.622 to 2.598) | 0.511 |
| I(log(WBC, 2)) | 1.138 | (1.08 to 1.2) | **<0.001** |
| FLT3I | 1.26 | (0.898 to 1.766) | 0.179 |
| NPM1 | 0.46 | (0.311 to 0.68) | **<0.001** |
| alSCTCR1 | 1.34 | (1.013 to 1.774) | **0.04** |
| FLT3I:NPM1 | 1.031 | (0.578 to 1.838) | 0.919 |

## Suppl. Table 7 (A) Univariate and (B) multivariate logistic regression model for CR1 in dm*CEBPA* bZIP^InDel^, sm*CEBPA* bZIP^InDel^ and *CEBPA*^other^.

## (A)

| **Parameter** | **OR** | **95%CI** | **p** |
| --- | --- | --- | --- |
| dm*CEBPA* bZIP^InDel^ | 5.82 | (3.618 to 9.363) | **<0.001** |
| sm*CEBPA* bZIP^InDel^ | 4.125 | (1.594 to 10.676) | **0.003** |

## (B)

| **Parameter** | **OR** | **95%CI** | **p** |
| --- | --- | --- | --- |
| dm*CEBPA* bZIP^InDel^ | 6.664 | (3.723 to 11.926) | **<0.001** |
| sm*CEBPA* bZIP^InDel^ | 4.432 | (1.631 to 12.044) | **0.004** |
| AGE | 0.974 | (0.959 to 0.99) | **0.001** |
| AMLSTATsAML | 0.483 | (0.222 to 1.049) | 0.066 |
| AMLSTATtAML | 0.612 | (0.16 to 2.342) | 0.473 |
| I(log(WBC, 2)) | 0.822 | (0.739 to 0.914) | **<0.001** |
| FLT3I | 0.566 | (0.293 to 1.091) | 0.089 |
| NPM1 | 2.122 | (1.072 to 4.2) | **0.031** |

## Suppl. Table 8 (A) Cox regression model and (B) multiple cox regression model for RFS in dm*CEBPA* bZIP^InDel^, sm*CEBPA* bZIP^InDel^ and *CEBPA*^other^.

## (A)

| **Parameter** | **HR** | **95%CI** | **p** |
| --- | --- | --- | --- |
| dm*CEBPA* bZIP^InDel^ | 0.517 | (0.421 to 0.634) | **<0.001** |
| sm*CEBPA* bZIP^InDel^ | 0.663 | (0.452 to 0.971) | **0.035** |

## (B)

| **Parameter** | **HR** | **95%CI** | **p** |
| --- | --- | --- | --- |
| dm*CEBPA* bZIP^InDel^ | 0.445 | (0.345 to 0.574) | **<0.001** |
| sm*CEBPA* bZIP^InDel^ | 0.52 | (0.338 to 0.799) | **0.003** |
| AGE | 1.021 | (1.013 to 1.029) | **<0.001** |
| AMLSTATsAML | 1.522 | (0.99 to 2.338) | 0.055 |
| AMLSTATtAML | 1.026 | (0.375 to 2.802) | 0.961 |
| I(log(WBC, 2)) | 1.146 | (1.083 to 1.212) | **<0.001** |
| FLT3I | 1.32 | (0.913 to 1.907) | 0.141 |
| NPM1 | 0.389 | (0.248 to 0.611) | **<0.001** |
| alSCTCR1 | 0.798 | (0.604 to 1.054) | 0.111 |

## Suppl. Table 9 (A) Cox regression model and (B) multiple cox regression model for OS in dm*CEBPA* bZIP^InDel^, sm*CEBPA* bZIP^InDel^ and *CEBPA*^other^.

## (A)

| **Parameter** | **HR** | **95%CI** | **p** |
| --- | --- | --- | --- |
| dm*CEBPA* bZIP^InDel^ | 0.347 | (0.281 to 0.429) | **<0.001** |
| sm*CEBPA* bZIP^InDel^ | 0.614 | (0.422 to 0.894) | **0.011** |

## (B)

| **Parameter** | **HR** | **95%CI** | **p** |
| --- | --- | --- | --- |
| dm*CEBPA* bZIP^InDel^ | 0.349 | (0.268 to 0.453) | **<0.001** |
| sm*CEBPA* bZIP^InDel^ | 0.48 | (0.313 to 0.737) | **0.001** |
| AGE | 1.03 | (1.022 to 1.038) | **<0.001** |
| AMLSTATsAML | 1.889 | (1.324 to 2.697) | **<0.001** |
| AMLSTATtAML | 1.281 | (0.63 to 2.607) | 0.493 |
| I(log(WBC, 2)) | 1.137 | (1.079 to 1.198) | **<0.001** |
| FLT3I | 1.253 | (0.899 to 1.747) | 0.181 |
| NPM1 | 0.463 | (0.319 to 0.672) | **<0.001** |
| alSCTCR1 | 1.341 | (1.015 to 1.774) | **0.038** |

## Suppl. Table 10 (A) Univariate and (B) multivariate cox regression model for OS in *CEBPA* bZIP^InDel^ with vs. without alloHCT in CR1 with interaction for study group. *P* for interaction test (A) =.77, (B) =.651

**(A)**

| **Parameter** | **HR** | **95%CI** | **p** |
| --- | --- | --- | --- |
| allo HCT | 1.252 | (0.707 to 2.219) | 0.441 |

**(B)**

| **Parameter** | **HR** | **95%CI** | **p** |
| --- | --- | --- | --- |
| alSCTCR1allo HCT | 1.528 | (0.852 to 2.74) | 0.155 |
| AGE | 1.041 | (1.026 to 1.056) | **<0.001** |
| AMLSTATsAML | 1.361 | (0.573 to 3.234) | 0.486 |
| AMLSTATtAML | 2.239 | (0.296 to 16.911) | 0.435 |
| I(log(WBC, 2)) | 1.187 | (1.068 to 1.32) | **0.001** |
| FLT3I | 0.65 | (0.363 to 1.162) | 0.147 |
| NPM1 | 0 | (0 to Inf) | 0.995 |
| (SGR)ALFA | 2.555 | (0.434 to 15.046) | 0.3 |
| (SGR)AMLCG | 0.753 | (0.141 to 4.034) | 0.741 |
| (SGR)HOVON | 0.978 | (0.317 to 3.014) | 0.969 |
| (SGR)MLL | 0.337 | (0.038 to 2.995) | 0.33 |
| (SGR)MRC | 0.478 | (0.096 to 2.388) | 0.369 |

## Suppl. Table 11 Multivariate cox regression model for (A) RFS and (B) OS in dm*CEBPA* bZIP^InDel^ with vs. without concomitant *CSF3R*^mut^.

**(A)**

| **Parameter** | **HR** | **95%CI** | **p** |
| --- | --- | --- | --- |
| dm*CEBPA* bZIP^InDel^, *CSF3R*^wt^ | 0.215 | (0.111 to 0.416) | **<0.001** |
| AGE | 1.018 | (1.005 to 1.03) | **0.003** |
| AMLSTATsAML | 1.041 | (0.327 to 3.31) | 0.946 |
| AMLSTATtAML | 3.024 | (0.412 to 22.221) | 0.277 |
| I(log(WBC, 2)) | 1.215 | (1.091 to 1.353) | **<0.001** |
| alSCTCR1 | 0.927 | (0.603 to 1.425) | 0.729 |

**(B)**

| **Parameter** | **HR** | **95%CI** | **p** |
| --- | --- | --- | --- |
| dm*CEBPA* bZIP^InDel^, *CSF3R*^wt^ | 1.983 | (0.485 to 8.102) | 0.341 |
| AGE | 1.039 | (1.023 to 1.055) | **<0.001** |
| AMLSTATsAML | 0.862 | (0.209 to 3.557) | 0.838 |
| AMLSTATtAML | 2.778 | (0.376 to 20.543) | 0.317 |
| I(log(WBC, 2)) | 1.177 | (1.043 to 1.328) | **0.009** |
| alSCTCR1 | 1.888 | (1.194 to 2.986) | **0.007** |

## Suppl. Table 12 Multivariate cox regression model for (A) RFS and (B) OS in dm*CEBPA* bZIP^InDel^ with vs. without concomitant *TET2*^mut^.

**(A)**

| **Parameter** | **HR** | **95%CI** | **p** |
| --- | --- | --- | --- |
| dm*CEBPA* bZIP^InDel^, *TET2*^wt^ | 0.722 | (0.452 to 1.154) | 0.174 |
| AGE | 1.017 | (1.003 to 1.031) | **0.015** |
| AMLSTATsAML | 1.048 | (0.329 to 3.341) | 0.937 |
| AMLSTATtAML | 0 | (0 to Inf) | 0.997 |
| I(log(WBC, 2)) | 1.184 | (1.057 to 1.326) | **0.004** |
| alSCTCR1 | 0.94 | (0.597 to 1.479) | 0.788 |

**(B)**

| **Parameter** | **HR** | **95%CI** | **p** |
| --- | --- | --- | --- |
| dm*CEBPA* bZIP^InDel^, *TET2*^wt^ | 0.755 | (0.435 to 1.309) | 0.317 |
| AGE | 1.039 | (1.022 to 1.057) | **<0.001** |
| AMLSTATsAML | 0.963 | (0.231 to 4.005) | 0.958 |
| AMLSTATtAML | 0 | (0 to Inf) | 0.998 |
| I(log(WBC, 2)) | 1.161 | (1.027 to 1.314) | **0.017** |
| alSCTCR1 | 2.037 | (1.248 to 3.324) | **0.004** |

## Suppl. Table 13 Multivariate cox regression model for (A) RFS and (B) OS in dm*CEBPA* bZIP^InDel^ with vs. without concomitant *FLT3*-ITD.

**(A)**

| **Parameter** | **HR** | **95%CI** | **p** |
| --- | --- | --- | --- |
| dm*CEBPA* bZIP^InDel^, no *FLT3*-ITD | 0.999 | (0.579 to 1.723) | 0.997 |
| AGE | 1.014 | (1.002 to 1.027) | **0.02** |
| AMLSTATsAML | 0.988 | (0.311 to 3.136) | 0.984 |
| AMLSTATtAML | 2.787 | (0.38 to 20.456) | 0.314 |
| I(log(WBC, 2)) | 1.191 | (1.073 to 1.322) | **0.001** |
| alSCTCR1 | 0.877 | (0.573 to 1.342) | 0.546 |

**(B)**

| **Parameter** | **HR** | **95%CI** | **p** |
| --- | --- | --- | --- |
| dm*CEBPA* bZIP^InDel^, no *FLT3*-ITD | 1.251 | (0.676 to 2.315) | 0.476 |
| AGE | 1.039 | (1.024 to 1.055) | **<0.001** |
| AMLSTATsAML | 0.853 | (0.207 to 3.515) | 0.827 |
| AMLSTATtAML | 2.749 | (0.372 to 20.305) | 0.322 |
| I(log(WBC, 2)) | 1.149 | (1.023 to 1.29) | **0.018** |
| alSCTCR1 | 1.768 | (1.122 to 2.786) | **0.014** |

## Suppl. Table 14 (A) Cox regression model and (B) multiple cox regression model for RFS in *CEBPA* bZIP^InDel^ with or without ELN2022 adverse.

**(A)**

| **Parameter** | **HR** | **95%CI** | **p** |
| --- | --- | --- | --- |
| ELN2022adv | 1.37 | (0.867 to 2.166) | 0.178 |

**(B)**

| **Parameter** | **HR** | **95%CI** | **p** |
| --- | --- | --- | --- |
| ELN2022adv | 1.227 | (0.76 to 1.981) | 0.405 |
| AGE | 1.018 | (1.004 to 1.032) | **0.01** |
| AMLSTATsAML | 1.209 | (0.522 to 2.798) | 0.657 |
| AMLSTATtAML | 0 | (0 to Inf) | 0.996 |
| I(log(WBC, 2)) | 1.167 | (1.051 to 1.294) | **0.004** |
| alSCTCR1 | 0.787 | (0.503 to 1.233) | 0.297 |

## Suppl. Table 15 (A) Cox regression model and (B) multiple cox regression model for OS in *CEBPA* bZIP^InDel^ with or without ELN2022 adverse.

**(A)**

| **Parameter** | **HR** | **95%CI** | **p** |
| --- | --- | --- | --- |
| ELN2022adv | 1.457 | (0.895 to 2.371) | 0.129 |

**(B)**

| **Parameter** | **HR** | **95%CI** | **p** |
| --- | --- | --- | --- |
| ELN2022adv | 1.202 | (0.712 to 2.029) | 0.491 |
| AGE | 1.036 | (1.02 to 1.053) | **<0.001** |
| AMLSTATsAML | 1.358 | (0.578 to 3.193) | 0.483 |
| AMLSTATtAML | 0 | (0 to Inf) | 0.996 |
| I(log(WBC, 2)) | 1.161 | (1.037 to 1.298) | **0.009** |
| alSCTCR1 | 1.459 | (0.905 to 2.35) | 0.121 |

## Suppl. Table 16 (A) Cox regression model and (B) multiple cox regression model for RFS in *CEBPA*^other^ with ELN2022 favorable, ELN2022 adverse or ELN2022 intermediate.

**(A)**

| **Parameter** | **HR** | **95%CI** | **p** |
| --- | --- | --- | --- |
| ELN2022fav | 0.496 | (0.313 to 0.787) | **0.003** |
| ELN2022adv | 1.202 | (0.848 to 1.703) | 0.301 |

**(B)**

| **Parameter** | **HR** | **95%CI** | **p** |
| --- | --- | --- | --- |
| ELN2022fav | 0.441 | (0.273 to 0.712) | **0.001** |
| ELN2022adv | 1.052 | (0.712 to 1.555) | 0.798 |
| AGE | 1.023 | (1.01 to 1.036) | **<0.001** |
| AMLSTATsAML | 1.522 | (0.908 to 2.552) | 0.112 |
| AMLSTATtAML | 0.755 | (0.235 to 2.432) | 0.638 |
| I(log(WBC, 2)) | 1.103 | (1.02 to 1.193) | **0.014** |
| alSCTCR1 | 0.835 | (0.544 to 1.282) | 0.411 |

## Suppl. Table 17 (A) Cox regression model and (B) multiple cox regression model for OS in *CEBPA*^other^ with ELN2022 favorable, ELN2022 adverse or ELN2022 intermediate.

**(A)**

| **Parameter** | **HR** | **95%CI** | **p** |
| --- | --- | --- | --- |
| ELN2022fav | 0.567 | (0.373 to 0.863) | **0.008** |
| ELN2022adv | 1.423 | (1.045 to 1.936) | **0.025** |

**(B)**

| **Parameter** | **HR** | **95%CI** | **p** |
| --- | --- | --- | --- |
| ELN2022fav | 0.551 | (0.357 to 0.85) | **0.007** |
| ELN2022adv | 1.18 | (0.841 to 1.657) | 0.337 |
| AGE | 1.027 | (1.015 to 1.039) | **<0.001** |
| AMLSTATsAML | 1.877 | (1.245 to 2.831) | **0.003** |
| AMLSTATtAML | 1.045 | (0.456 to 2.394) | 0.917 |
| I(log(WBC, 2)) | 1.111 | (1.038 to 1.19) | **0.002** |
| alSCTCR1 | 1.271 | (0.842 to 1.921) | 0.255 |

## Suppl. Figure 1 Kaplan–Meier estimates of OS in *CEBPA* bZIP^InDel^ patients with vs. without alloHCT performed in CR1. *P*value was calculated by Cox regression model.


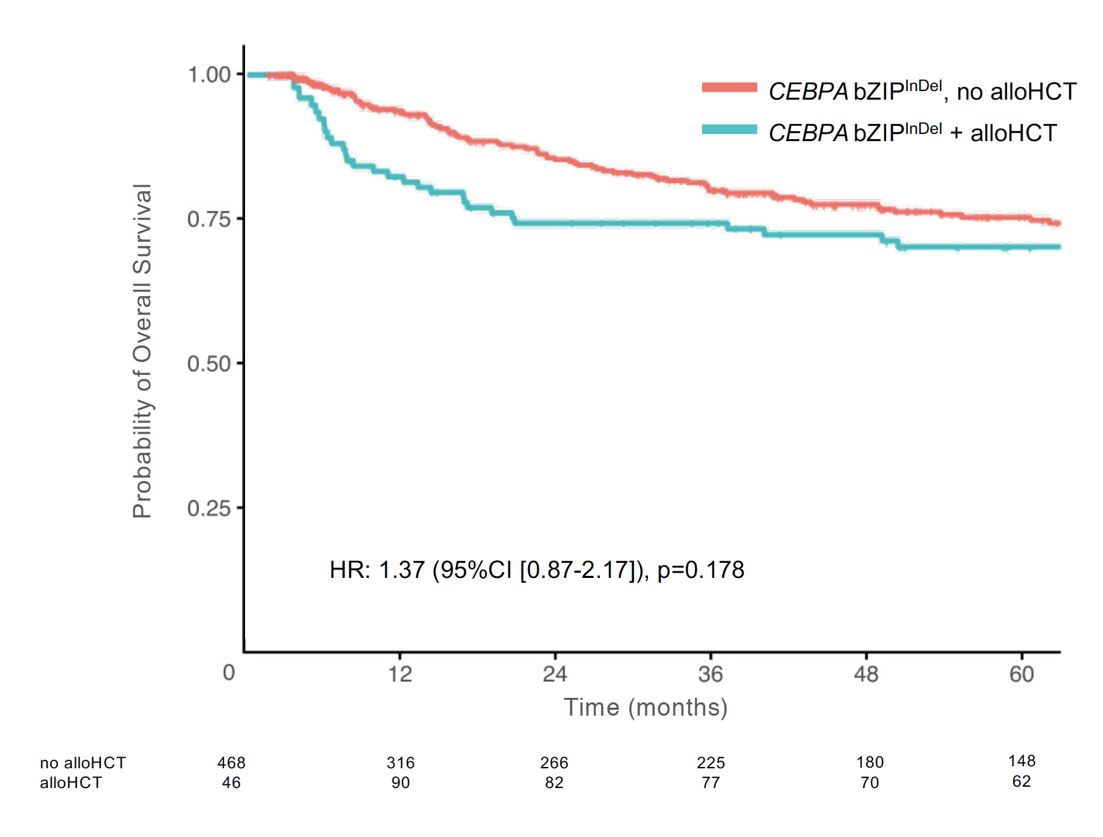


## Suppl. Figure 2 Kaplan–Meier estimates of (A) RFS and (B) OS in dm*CEBPA* bZIP^InDel^ patients with vs. without *CSF3R*^mut^. *P*value was calculated by Cox regression model.


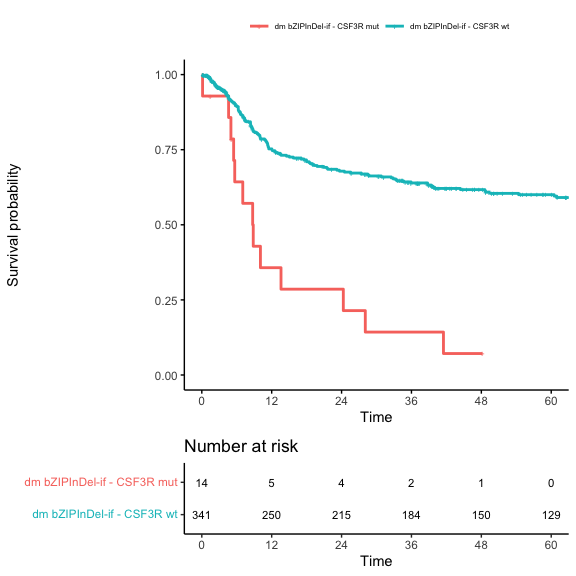
**(A)** **(B)**

Probability of Overall Survival

Probability of Relapse-free Survival


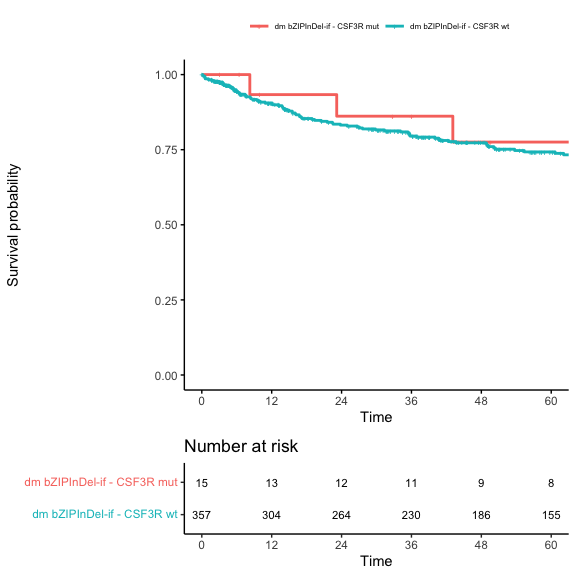


*p*=.491

***p*<.001**

dm*CEBPA*bZIP^InDel^, *CSF3R*^wt^

dm*CEBPA*bZIP^InDel^, *CSF3R*^mut^

dm*CEBPA*bZIP^InDel^, *CSF3R*^wt^

dm*CEBPA*bZIP^InDel^, *CSF3R*^mut^

## Suppl. Figure 3 Kaplan–Meier estimates of (A) RFS and (B) OS in dm*CEBPA* bZIP^InDel^ patients with vs. without *TET2*^mut^. *P*value was calculated by Cox regression model.

###
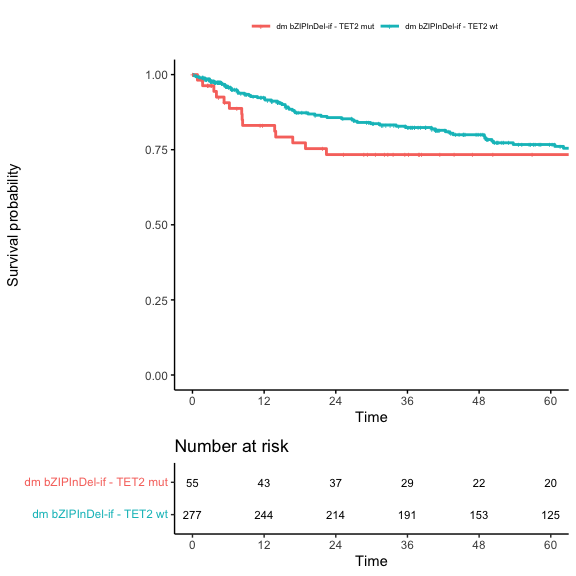

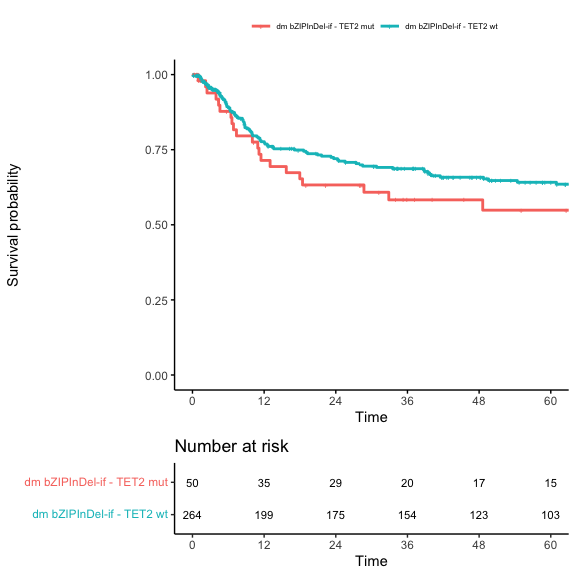
(A) (B)

dm*CEBPA*bZIP^InDel^, *TET2*^mut^

dm*CEBPA*bZIP^InDel^, *TET2*^wt^

dm*CEBPA*bZIP^InDel^, *TET2*^wt^

dm*CEBPA*bZIP^InDel^, *TET2*^mut^

Probability of Overall Survival

Probability of Relapse-free Survival

***p*=.025**

***p*=.028**

## Suppl. Figure 4 Kaplan–Meier estimates of (A) RFS and (B) OS in dm*CEBPA* bZIP^InDel^ patients with vs. without *FLT3*-ITD. *P*value was calculated by Cox regression model.

###
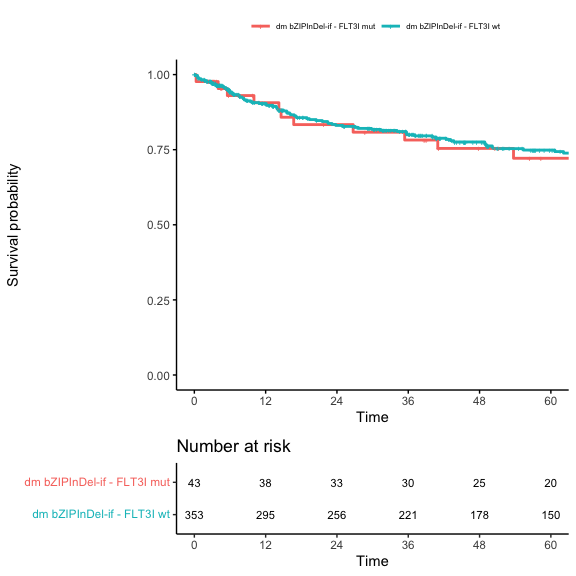
A) (B)


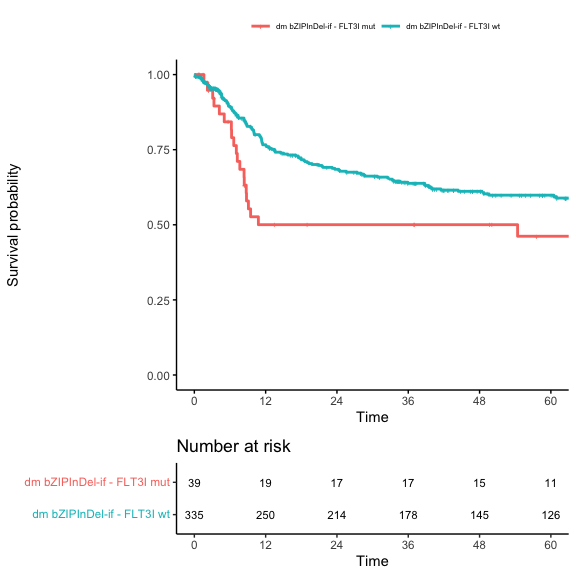


dm*CEBPA*bZIP^InDel^, *FLT3-*ITD^pos^

dm*CEBPA*bZIP^InDel^, no *FLT3*^-ITD^

dm*CEBPA*bZIP^InDel^, no *FLT3*^-ITD^

dm*CEBPA*bZIP^InDel^, *FLT3-*ITD^pos^

Probability of Overall Survival

Probability of Relapse-free Survival

***p*=.031**

*p*=.233

## Suppl. Figure 5 Kaplan–Meier estimates of (A) RFS and (B) OS in dm*CEBPA* bZIP^InDel^ patients with vs. without *GATA2*^mut^. *P*value was calculated by Cox regression model.


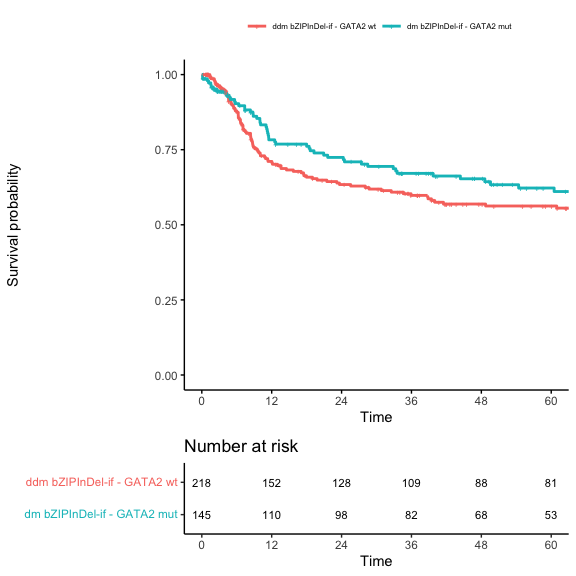

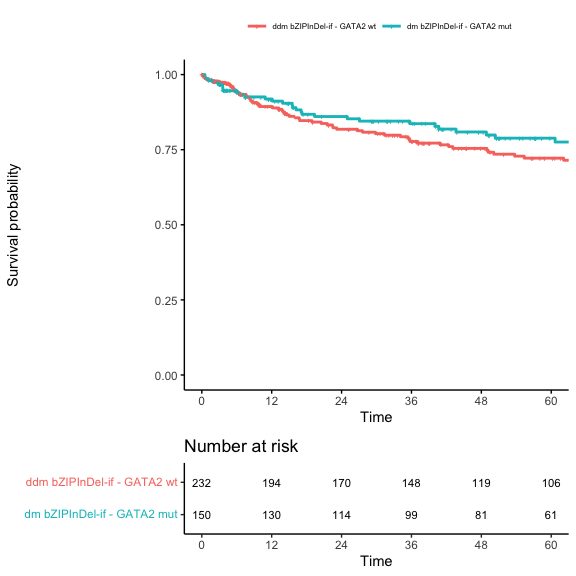
**(A)** **(B)**

*p*=.52

Probability of Overall Survival

Probability of Relapse-free Survival

*p*=.145

*p*=.075

dm*CEBPA*bZIP^InDel^, *GATA2*^mut^

dm*CEBPA*bZIP^InDel^, *GATA2*^wt^

dm*CEBPA*bZIP^InDel^, *GATA2*^wt^

dm*CEBPA*bZIP^InDel^, *GATA2*^mut^

## Suppl. Figure 6 Kaplan–Meier estimates of (A) RFS and (B) OS in dm*CEBPA* bZIP^InDel^ patients with vs. without *WT1*^mut^. *P*value was calculated by Cox regression model.


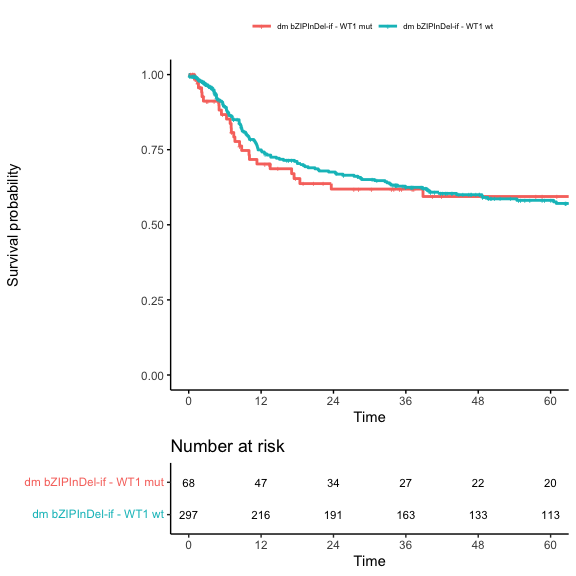

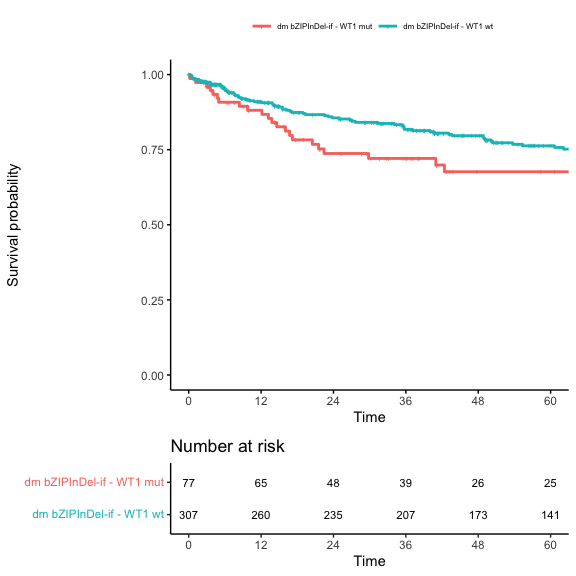
**(A)** **(B)**

Probability of Relapse-free Survival

Probability of Overall Survival

*p*=.173

*p*=.906

dm*CEBPA*bZIP^InDel^, *WT1*^wt^

dm*CEBPA*bZIP^InDel^, *WT1*^mut^

dm*CEBPA*bZIP^InDel^, *WT1*^wt^

dm*CEBPA*bZIP^InDel^, *WT1*^mut^

## Description of clinical trial protocols and numbers of treated patients

**AML96 (n=98)** (NCT00180115): Risk-adapted postremission treatment strategy including related and unrelated allogeneic HCT for high risk AML patients and related allogeneic and autologous HCT for standard risk AML patients. Randomization between intermediate-dose Cytarabine and high-dose Cytarabine within the first postremission-course.

**AML60+ (n=14)** (NCT00180167): Randomization between DA and intermediate-dose cytarabine plus mitoxantrone for induction in AML patients >=60 yrs of age.

**AML2003 (n=58)** (NCT00180102): Risk-adapted post-remission therapy including allogeneic HCT in aplasia or in CR1 and autologous HCT in AML patients. Randomization between three cycles of high-dose cytarabine and MAC-MAMAC-MAC for postremission therapy.

**SORAML (n=11)** (NCT00893373): Randomization between addition of either sorafenib or placebo to standard treatment in AML patients (consisting of DA followed by high-dose cytarabine consolidation therapy or alloHCT for intermediate-risk patients with a sibling donor and for all high-risk patients with an HLA-matched donor in first remission).

**HO29 AML (n=16)** (ISRCTN76815071): Remission Induction Chemotherapy with or without rHuG-CSF (lenograstim) sensitization. Marrow Ablative Chemotherapy plus autologous HCT versus Chemotherapy in AML patients.

**HO42 AML (n=29)** (ISRCTN38648181)**:** Randomized induction and post induction therapy in adult patients (<= 60 yrs of age) with AML or refractory anemia with excess of blasts (RAEB, RAEB-t).

**HO43 AML (n=4)** (ISRCTN77039377): Randomized induction and post induction therapy in older patients (>= 61 yrs of age) with AML and RAEB, RAEB-t.

**HO81 AML (n=7)** (ISRCTN18332222): Tolerability and efficacy of the addition of Bevacizumab to standard induction therapy in AML and high risk MDS above 60 yrs.

**HO92 AML (n=14)** (NL1386): Randomized study to assess the added value of Laromustine in combination with standard remission-induction chemotherapy in patients aged 18-65 yrs with previously untreated AML or MDS (RAEB with IPSS ≥ 1.5).

**HO102 AML (n=18)** (NL2070): Randomized study with a run-in feasibility phase to assess the added value of Clofarabine in combination with standard remission-induction chemotherapy in patients aged 18-65 yrs with previously untreated AML or MDS (RAEB with IPSS ≥ 1.5).

**HO103 AML (n=33)** (NL2370): Tolerability and efficacy of the addition of new drugs to standard induction chemotherapy in AML and high-risk myelodysplasia (MDS) (IPSS-R risk score > 4.5) in patients aged >= 66 yrs.

**HO132 AML (n=58)** (NL4231): Randomized study with a run-in dose-selection phase to assess the added value of lenalidomide in combination with standard remission-induction chemotherapy and post-remission treatment in patients aged 18-65 yrs with previously untreated AML or high risk MDS (IPSS-R risk score > 4.5).

**AMLCG99 (n=64)** (NCT00266136): Risk-stratified randomization between standard treatment (double induction with TAD and HAM, consolidation with TAD) and double induction with HAM-HAM, multiple course G-CSF before and during chemotherapy courses and autologous HCT.

**AMLCG2008 (n=21) (**NCT01382147): Randomization between "dose-dense therapy" by S-HAM and double induction with TAD-HAM for remission induction in patients with AML.

**ALFA-1200 (n=29)** (NCT01966497): Observational study of patients older than 60 yrs with AML who are administered standard chemotherapy based on Idarubicine-cytarabine.

**ALFA-0701 (n=17)** (NCT00927498): Multicentric randomization between the combination of repeated doses of Gemtuzumab Ozogamicin (GO) with Daunorubicin and Cytarabine versus Daunorubicin and Cytarabine in untreated patients with AML aged 50-70 yrs.

**ALFA-0702 (n=53)** (NCT00932412): Randomization of Clofarabine/intermediate-dose Cytarabine (CLARA) versus high-dose Cytarabine (HDAC) as consolidation in younger patients with newly diagnosed AML.

**MRC-AML10 (n=41)** (DOI: 10.1016/s0140-6736(97)09214-3): Randomised comparison of addition of autologous bone-marrow transplantation to intensive chemotherapy for AML in first remission.

**MRC-AML12 (n=69)** (ISRCTN55678797): Randomized evaluation of (1) standard dose versus higher dose (Ara-C within a DAT (daunorubicin, Ara-C, thioguanine) induction regimen, (2) addition of retinoic acid (ATRA) during and after induction chemotherapy, (3) four versus five courses of therapy in total (where the final course is either chemotherapy or transplant), (4) bone marrow transplantation (either allogenic or autologous) versus conventional chemotherapy as the final course.

**MRC-AML15 (n=68)** (ISRCTN17161961): Comparison of (1) two induction schedules (DAT and FLAG-Ida), (2) standard consolidation chemotherapy (i.e. MACE + MidAC) versus high-dose Ara-C, (3) high-dose ARA-C during consolidation at two different doses, (4) four versus five courses of treatment in total (where the final course is intermediate-dose Ara-C), (5) assessment of the value of Mylotarg during induction and consolidation, (6) the value of allogeneic stem cell transplantation.

The remaining 288 patients were treated outside of clinical trial protocols with intensive (age-adjusted) regimens with curative intent according to local standards.
